# Dosing interval strategies for two-dose COVID-19 vaccination in 13 middle-income countries of Europe: Health impact modelling and benefit-risk analysis

**DOI:** 10.1016/j.lanepe.2022.100381

**Published:** 2022-04-11

**Authors:** Yang Liu, Carl A.B. Pearson, Frank G. Sandmann, Rosanna C. Barnard, Jong-Hoon Kim, CMMID COVID-19 Working Group, Stefan Flasche, Mark Jit, Kaja Abbas

**Affiliations:** a Centre for Mathematical Modelling of Infectious Diseases, London School of Hygiene & Tropical Medicine, London, United Kingdom; b Department of Infectious Disease Epidemiology, Faculty of Epidemiology and Population Health, London School of Hygiene & Tropical Medicine, London, United Kingdom; c Statistics, Modelling and Economics Department, National Infection Service, UK Health Security Agency (UK HSA), London, United Kingdom; d International Vaccine Institute, Seoul, South Korea

**Keywords:** Vaccine policy, Mathematical modelling, COVID-19, SARS-CoV-2, Public health intervention, Quantitative methods, MIC, Middle income country, AEFI, Adverse events following immunisation, VOC, Variant of Concern, VE, Vaccine Efficacy

## Abstract

**Background:**

In settings where the COVID-19 vaccine supply is constrained, extending the intervals between the first and second doses of the COVID-19 vaccine may allow more people receive their first doses earlier. Our aim is to estimate the health impact of COVID-19 vaccination alongside benefit-risk assessment of different dosing intervals in 13 middle-income countries (MICs) of Europe.

**Methods:**

We fitted a dynamic transmission model to country-level daily reported COVID-19 mortality in 13 MICs in Europe (Albania, Armenia, Azerbaijan, Belarus, Bosnia and Herzegovina, Bulgaria, Georgia, Republic of Moldova, Russian Federation, Serbia, North Macedonia, Turkey, and Ukraine). A vaccine product with characteristics similar to those of the Oxford/AstraZeneca COVID-19 (AZD1222) vaccine was used in the base case scenario and was complemented by sensitivity analyses around efficacies similar to other COVID-19 vaccines. Both fixed dosing intervals at 4, 8, 12, 16, and 20 weeks and dose-specific intervals that prioritise specific doses for certain age groups were tested. Optimal intervals minimise COVID-19 mortality between March 2021 and December 2022. We incorporated the emergence of variants of concern (VOCs) into the model and conducted a benefit-risk assessment to quantify the tradeoff between health benefits versus adverse events following immunisation.

**Findings:**

In all countries modelled, optimal strategies are those that prioritise the first doses among older adults (60+ years) or adults (20+ years), which lead to dosing intervals longer than six months. In comparison, a four-week fixed dosing interval may incur 10.1% [range: 4.3% - 19.0%; n = 13 (countries)] more deaths. The rapid waning of the immunity induced by the first dose (i.e. with means ranging 60-120 days as opposed to 360 days in the base case) resulted in shorter optimal dosing intervals of 8-20 weeks. Benefit-risk ratios were the highest for fixed dosing intervals of 8-12 weeks.

**Interpretation:**

We infer that longer dosing intervals of over six months could reduce COVID-19 mortality in MICs of Europe. Certain parameters, such as rapid waning of first-dose induced immunity and increased immune escape through the emergence of VOCs, could significantly shorten the optimal dosing intervals.

**Funding:**

World Health Organization.

**Research in context**

**Evidence before this study**

We searched PubMed with the search terms “(COVID-19 OR coronavirus OR SARS-CoV-2) AND (vaccine) AND (delay second dose OR late second dose)” on 8 November 2021 with no language restrictions. This returned 89 articles, of which 16 articles were relevant to inferring the health impact of different dosing intervals in high-income country settings. Based on the evidence synthesis of the relevant articles, we infer that optimal timing of dosing intervals for two-dose COVID-19 vaccines depends on multiple factors, including pre-existing naturally acquired immunity, serological response after first and second doses, vaccine efficacy and effectiveness after first and second doses, waning dynamics of vaccine-induced immunity, vaccination coverage, vaccine supply rates, variants of concern characteristics and country-specific vaccine prioritisation plans.

**Added value of this study**

To the best of our knowledge, this is the first health impact and benefit-risk assessment study of dosing interval strategies for two-dose COVID-19 vaccination outside of high-income settings. We assessed the health impact of COVID-19 vaccination for strategies using different dosing interval approaches after fitting to the COVID-19 mortality data for 13 middle income countries of Europe. We found that the strategy of prioritising the first dose coverage in older adults or adults was the optimal strategy to minimise COVID-19 mortality. Specific variants or vaccine characteristics, such as faster waning duration of the first dose, could significantly modify this conclusion – in which case, fixed dosing intervals between 8 and 20 weeks would be optimal.

**Implications of all the available evidence**

The optimal dosing intervals for two-dose COVID-19 vaccines are context-specific, depend on the underlying SARS-CoV-2 epidemiology in each country, and are sensitive to variants and vaccine characteristics. We showed that increasing the first dose coverage is key to minimising COVID-19 mortality. However, new evidence on parameters such as variants of concern's transmissibility and severity and (vaccine- or infection-induced) protection waning rate should be incorporated into consideration as they emerge.

Alt-text: Unlabelled box

## Introduction

By October 2021, 22 COVID-19 vaccines were in use globally.^1^ However, vaccine supply has struggled to meet the global demand. Low- and middle-income countries (LMICs) faced delays in vaccine roll-out. These constraints are expected to ease in late 2022 as additional production capacity becomes available.^2^ In the interim, it is vital that countries can maximise the health impact of the available vaccine supplies based on context-specific COVID-19 epidemiology and SARS-CoV-2 transmission dynamics, pre-existing immunity, and COVID-19 vaccine safety, immunogenicity, and efficacy.^3^

Most of the available vaccines involve two doses with recommended between-dose intervals as tested in clinical trials. These dosing intervals are generally 3-4 weeks, although they may differ by vaccine product. However, in practice, countries may use dosing intervals longer than recommended due to a wide range of factors, including but not limited to administrative and logistic constraints (e.g. vaccine clinic capacity), vaccine shortages, and the comparable and potentially higher vaccine efficacy using certain extended dosing intervals.^4^ Particularly, countries need to consider the tradeoff between partial protection (induced by one dose) of a larger number of individuals versus full protection (induced by two doses) of fewer individuals.

However, existing evidence on these tradeoffs has been limited and almost exclusively based on high-income settings.^5^ Additionally, such evidence is either not based on observed epidemic history^6^, ^7^, ^8^, ^9^, ^10^ (and thus do not incorporate realistic existing immunity level, which is crucial to vaccine strategies) or only based on epidemic history in high-income settings^11^; have not considered comprehensive vaccine effect mechanisms (e.g., prevention of onward-transmission) or immune dynamics (e.g., the potential waning after the first dose)^6^, ^7^, ^8^^,^^10^^,^^11^; have not accounted for the effects of variants of concern (VOCs) emergence^6^, ^7^, ^8^, ^9^, ^10^, ^11^; or have only tested for resource-abundant vaccine supply conditions applicable to high-income settings.^7^^,^^8^

Moreover, there are concerns about the benefits of the COVID-19 vaccine versus the harms from adverse events following immunisation (AEFI) depending on the age of the vaccinated individuals, which may change conclusions of the overall health benefits of vaccination.

The World Health Organisation commissioned this work to specifically generate country-level evidence outside of high-income settings to address this evidence gap. We used a mathematical modelling approach that incorporated two-dose dynamics to assess the health impacts (i.e. COVID-19 mortality) of different dosing intervals in middle-income countries (MICs) in Europe between March 2021 and December 2022. We aim to identify the optimal dosing interval strategy that minimises cumulative mortality or maximises the benefit-risk ratio. The evidence generated could inform COVID-19 vaccine policies as vaccines roll out - rapid shifts in policies have been shown feasible.^12^ As the MICs in Europe represent a wide range of population age structures and epidemic histories, the evidence could be valuable to vaccine policies in LMICs elsewhere in the world facing similar issues.

We explored country-level dosing interval policy approaches: (1) by setting fixed dosing intervals of different lengths; (2) by setting coverage targets (e.g. to first cover the entire adult population with the first dose). In the baseline scenario, we based the vaccine characteristics on those of the Oxford/AstraZeneca COVID-19 vaccine AZD1222 (hereafter AZD1222, which accounts for the largest share in the COVAX initiative that aims at accelerating global access to COVID-19 vaccines).^13^ We followed up with extensive sensitivity analyses for vaccine characteristics similar to other COVID-19 vaccines.

## Methods

### The mathematical modelling process

We adapted CovidM, an existing transmission dynamic model of SARS-CoV-2, which has already been extensively described elsewhere,^14^, ^15^, ^16^, ^17^, ^18^, ^19^ to estimate the health impact of COVID-19 vaccination. We attach the complete parameter table (relevant for the entire Methods) and their references in Supplemental Table S1.

**Figure 1:**
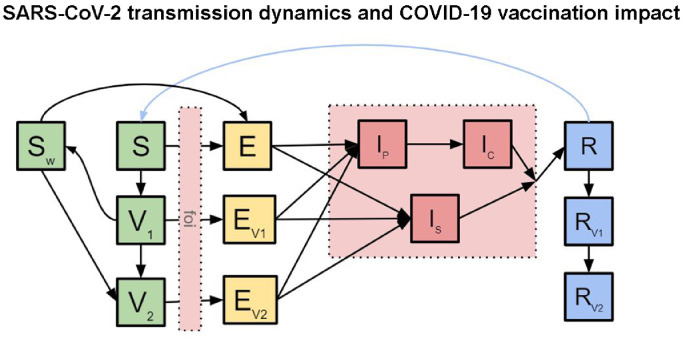
The conceptual diagram describes the underlying mathematical models of SARS-CoV-2 transmission dynamics and COVID-19 vaccination impact. S - susceptible; V_1_ - individuals protected by the first dose only; V_2_ - individuals protected by both doses; S_w_ - individuals who have received their first dose but the protection has waned; E - exposed; E_v1_ - exposed progressed from individuals in V_1_; E_v2_ - exposed progressed from individuals in V_2_; I_p_ - pre-clinical infectious individuals; I_c_ - clinical individuals; I_s_ - subclinical individuals; R - recovered; R_v1_ - previously infected individuals whose infection-induced immunity has yet to wane and who have received the first dose; R_v2_ individuals whose infection-induced immunity has yet to wane and who have received both doses.

In brief, CovidM is an SEIR-type (susceptible, exposed, infectious, and recovered) compartmental model consisting of 16 age groups (0-4 to 75+ years with five-year increments) to incorporate age-specific susceptibility, clinical fractions, population sizes, and contact patterns. Thirteen compartments were used to capture the population dynamics within and between age group (Figure 1).

Compared to a classic SEIR model, this framework has several unique features. First, individuals protected by one and two doses are modelled separately (V_1_ and V_2_ in Figure 1), allowing for the incorporation of dose-specific dynamics. Second, as evidence suggests,^4^^,^^20^ we accounted for potential waning among those who have only received their first doses (Sw in Figure 1). Leaving this element out would introduce a strong bias for longer dosing intervals. Third, recovered individuals could receive vaccinations (R_v1_ and R_v2_ in Figure 1), which could help us explore how the existing prevalence of infection-induced immunity affect the optimal vaccine dosing strategy. In Europe, antibody tests have not been used to qualify individuals for vaccination. Some evidence suggests that previously infected individuals may have higher neutralising antibody responses.^21^ However, the model has left out breakthrough infections among those with infection and vaccination history for dose accounting purposes (i.e. making sure all individuals receive 0-2 doses). Fourth, to account for the significant deviation from the public's pre-pandemic routine, we used Google Community Mobility Index (hereafter "mobility index") to adjust the contact matrices.^22^ This scaling method has been previously presented elsewhere.^23^ A brief description can be found in the Supplemental Methods p22.

This model was fitted to the country-level daily reported COVID-19 deaths before March 2021 in 13 MICs in Europe: Albania, Armenia, Azerbaijan, Belarus, Bosnia and Herzegovina, Bulgaria, Georgia, Republic of Moldova, Russian Federation, Serbia, North Macedonia, Turkey, and Ukraine for three variables: infection introduction date, basic reproduction number, and under-reporting rate. Among these variables, the under-reporting rate is particularly relevant outside of high-income settings due to potential testing and reporting capacity constraints.

Country-specific fitted results can be found in the GitHub repository attached to this study. We did not fit the model to the remaining seven LMICs with the same WHO Region due to data availability (n = 1), data sparsity issues (<10 deaths/ day throughout the fitting period, n = 4) or significant changes in ways tallying COVID-19 mortality (n = 2). Country characteristics are captured by population age structures, age-specific contact patterns, changes in the mobility index, epidemic history, and immunity levels before vaccine introduction.

The objective of the fitting process is to reproduce daily COVID-19 mortality time-series by country, assuming each observed daily COVID-19 mortality count is sampled from an underlying Poisson distribution. The final model selected maximised the overall likelihood of the entire fitting window. The parameter set associated with the optimal model was identified using differential evolution algorithms for each country.^24^ More details on the fitting process have been described in detail elsewhere.^23^

Given this study's relatively short time horizon, we modelled COVID-19 severe cases and mortality as processes, apart from the population dynamics presented in Figure 1 (i.e., there is no removal of individuals from the modelled population). The total COVID-19 severe case incidence and mortality is the product of age-specific infection counts, infection-hospitalisation risks/infection-fatality risks, and functions describing the delay between infection and hospitalisation/ mortality. Equations describing these processes can also be found in the Supplemental Methods p23.

### Vaccine characteristics

**Figure 2:**
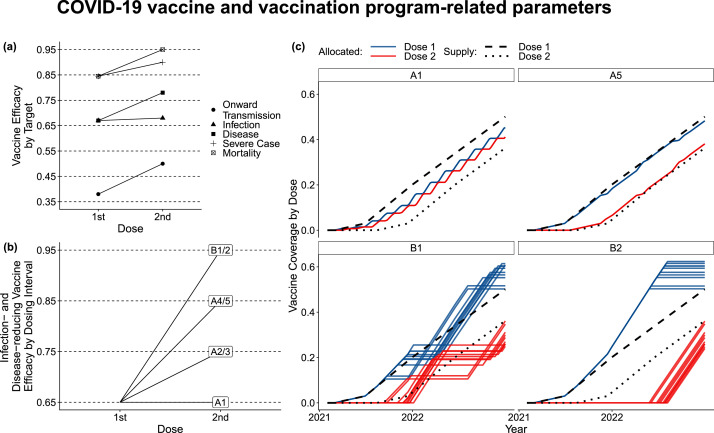
(a) Vaccine efficacy by effect type, respectively describing onward transmission blocking, infection-, disease-, severe case- and mortality-reducing vaccine efficacies. (b) Sensitivity analysis parameter set that describes a condition where longer dosing interval is associated with larger incremental change incurred by the second doses in infection- and disease-reducing vaccine efficacies. (c) Vaccine supply and allocation conditions by dose. We assume a 24-week delay between the supply of first and second doses. We did not show strategies A2, A3 and A4 as they reflect incremental changes between A1 and A5. Each line represents a country. Refer to Table 1 for descriptions of vaccination strategies.

In the base case scenario, we considered a vaccine product with characteristics similar to AZD1222 and incorporated five types of vaccine effects (i.e. infection-, disease-, severe case-, mortality-, and onward transmission-reducing) into the model (Figure 2, a). For the rationale behind specific parameters used, please refer to Supplemental Table S3.^18^

Given the substantial uncertainty around the estimates of vaccine efficacies and the possibility that a country may use other (or multiple) vaccine products, we conducted sensitivity analyses over four dimensions around the values for infection- and disease-reducing vaccine efficacies for the first and second doses (Supplemental Methods p24). This parameter space account for our best understanding of all vaccine products available (e.g., mRNA vaccines). There is also limited evidence that suggests higher vaccine efficacy after the second dose when the dosing interval is longer than label recommendations.^20^ We explored how this may affect the optimal dosing strategy (Figure 2, b).

We implemented a 14-day delay between vaccination (i.e. doses administered) and immunisations (i.e. protection developed).

There is evidence showing the potential waning of the first dose when the second dose has not been administered promptly.^4^^,^^20^ In this study, we assume this waning process (loss of protection from the vaccine) follows exponential decay with an average duration of 360 days. If this waning process occurs faster than expected (e.g., due to VOC emergence), a shorter waning duration of 120 days was tested as a worst-case scenario sensitivity analysis. Additional sensitivity analyses tested even shorter waning durations as characteristics of future VOC are largely uncertain.

### Strategies regarding dosing intervals

This study used two approaches to set dosing intervals (Table 1) as some countries may choose to fix an *n* week dosing interval between the first and second doses for all individuals while others may aim to cover all individuals with the first dose before vaccinating anyone with the second dose. We expanded these two general approaches into seven dosing interval strategies (Figure 2, c). We assume protection following the first dose may wane before the second dose is given; protection following the second dose, however, is assumed not to wane regardless of the dosing interval.

**Table 1:** Vaccination strategies.

Strategy	Description
A1	Individuals will receive their 2nd doses ***4*** weeks after their first doses.
A2	Individuals will receive their 2nd doses ***8*** weeks after their first doses.
A3	Individuals will receive their 2nd doses ***12*** weeks after their first doses.
A4	Individuals will receive their 2nd doses ***16*** weeks after their first doses.
A5	Individuals will receive their 2nd doses ***20*** weeks after their first doses.
B1	Older adults will receive their 2nd doses after all older adults receive their first doses. Young adults will receive their 1st doses after older adults uptake targets are reached. Young adults will only receive 2nd doses when all young adults have received their 1st doses.
B2	Older adults will receive their 2nd doses after all individuals (older adults + young adults) receive their first doses; young adults will receive their 2nd doses after older adults uptake targets are reached, and all young adults have received their first dose

Definitions of vaccine dosing strategies. Older adults: those above 60 years of age; young adults: those between 20 and 59 years of age.

### Other assumptions around the vaccination programmes

We have previously shown that an age-based vaccine prioritisation strategy that targets older adults first (i.e. 60+) and then moves on to young adults (i.e. 20–59) consistently performs comparably or better than other age-based vaccine prioritisation strategies.^23^ All dosing intervals we tested were in addition to this age-based vaccine prioritisation strategy.

We additionally assume the uptake goal for older adults is 90% and for young adults 70%. In other words, when the uptake level has reached 90% among those above 60 years, the vaccination program is considered complete for that age group and will move on to vaccinating adults. These two uptake thresholds are feasible based on age-specific uptake levels in countries with relatively fast vaccine roll-out in older adults.

We assumed the starting date of vaccination programs to be 01 March 2021 in LMICs from WHO press releases. Based on the COVAX vaccine supply forecast to inform vaccine supply,^2^ we set the vaccine supply targets of the first doses to be 3% of the total population by mid-2021 and 20% of the total population by the end of 2021. With a slightly faster rate, we assume this program will cover 50% of the population with their first dose by the end of 2022. In this study, we assume that the rate of vaccine supply captures both the availability of vaccine doses and limits on the resources needed to deliver the vaccine doses to vaccinees which constrain the speed of vaccine roll-out.

The roll-out timelines specified above are consistent with the slowest adaptors among MICs considered (Supplemental Fig. S1). With these vaccination objectives, we explored the difference between strategies B1 and B2. When vaccine supply is much lower than explored here, B1 and B2 may lead to the same results as their algorithms only diverge after all older adults have been vaccinated with the first doses.

To investigate the impacts of vaccine supply delay, we assume the second dose will become available 24 weeks after their initial inoculation dates. As sensitivity analyses, we also explored supply delay levels at 12 and 52 weeks.

As vaccines roll out, contact levels within the population may recover. This study assumes contacts gradually recover to near pre-pandemic levels (i.e. 90% recovery) over a year from March 2021 following a sigmoid function. We do not account for reactive public health and social measures in response to surging infections as the action threshold (i.e. the definition of "surging infections") and the action intensity (e.g. a lockdown or a face mask mandate) may vary significantly by country.

### Consideration around variants of concern

The emergence of VOCs may reduce the efficacy of vaccines. We investigated the potential effects of VOC emergence by modifying disease dynamics and vaccine efficacy. To reflect the impact of variants, we applied a 1.5x multiplier to transmissibility.^16^^,^^18^^,^^25^ This effectively increases the reproduction number by 50% while other factors that influence the realized effective reproduction number (e.g. contacts) remain constant. To capture the potential increase in severity, we implemented 50% increases in infection fatality and hospitalisation ratios regardless of vaccination status. We included a 40% reduction in infection-reducing vaccine efficacy to account for potential immune escape. This reduction in efficacy may also be interpreted as the rapid loss of infection-reducing protection after a potential VOC introduction. Other vaccine effect pathways (i.e. disease-, severe cases- mortality-, onward transmission-reducing) were assumed to remain unchanged despite VOC emergence. These characteristics have been assumed based on current evidence around the Delta variant.^18^^,^^26^ All changes described were introduced into the simulation processes on 15 April 2021 simultaneously, broadly aligning with the approximate timing of the large-scale emergence of the Delta variant in western Europe.^27^^,^^28^

### Criteria for optimal dosing strategy

We used the cumulative mortality between 01 March 2021 and 31 December 2022 COVID-19 as the primary decision-making metric. The optimal dosing interval strategy minimises the cumulative mortality. As discussed above, COVID-19 mortality is the product of age-specific infection counts, infection-fatality ratios, and a temporal delay function representing the interval between becoming infected and dying of COVID-19 (Supplemental Methods p23). We compare dosing interval strategies by calculating the percentage difference in cumulative mortality relative to the reference strategy B1.

### Benefit-risk analysis of COVID-19 vaccination versus adverse events following immunisation

In line with the previous parameterisation based on AZD1222, we quantified the risk of fatal AEFI based on major thromboembolic events (blood clots) with low platelet count (thrombocytopenia) as reported for the AZD1222. In the UK, up to 01 September 2021, there have been 416 cases in total, of which 45 cases occurred after the second dose.^29^ A total of 72 deaths occurred, with 6 deaths reported after the second dose. Given the different number of doses given by age in the UK, the age-specific gradient of the risk of developing these serious AEFIs is about 20.5 per 1 million doses in individuals aged 18–49 years and 10.9 per 1 million doses in individuals aged ≥ 50 years after the first dose; and 0.9 per 1 million doses and 1.9 per 1 million doses after the second dose, respectively.^29^

Based on the results of the different dosing intervals in the main analysis, we used these age-specific rates of occurrence after the first and second dose, respectively, times the proportion of fatalities after the first and second dose to estimate the total number of fatal AEFIs based on the number of vaccine doses used in each country.

We then traded off the age-specific mortality from COVID-19 versus the age-specific mortality caused by AEFIs. We took the worst-performing dosing strategy in terms of mortality as the baseline to estimate the benefit-risk ratios, which quantify the benefit of additional deaths prevented by the vaccines versus the harm from additional deaths caused by AEFIs. For this analysis, strategy A1 is taken as the baseline to obtain positive results for the benefits (and the benefit-risk ratios) to facilitate interpretation. If one was to consider a no-vaccination strategy (which is highly unlikely for COVID-19 in general, and in particular for the countries included here), then all of the benefit-risk ratios against no-vaccination are expected to be greater than 1.0 given the estimated much larger rate of outcomes in SARS-CoV-2 positive cases among unvaccinated individuals than vaccinated individuals.^30^

All the program code and data used in the study are publicly accessible online at https://github.com/yangclaraliu/COVID_Vac_Delay.

### Role of the funding source

The funders were involved in study design, data collection, data analysis, data interpretation, writing of the report, and the decision to submit for publication. We had discussions and received feedback from the members of the WHO Strategic Advisory Group of Experts on Immunization (SAGE) Working group on COVID-19 vaccine impact modelling and Immunisation, and Vaccines related Implementation Research Advisory Committee (IVIR-AC). All authors had full access to all the data in the study and had final responsibility for the decision to submit for publication. This study was approved by the ethics committee (Ref 26318) of the London School of Hygiene & Tropical Medicine.

## Results

### The implication of different dosing interval strategies

**Figure 3:**
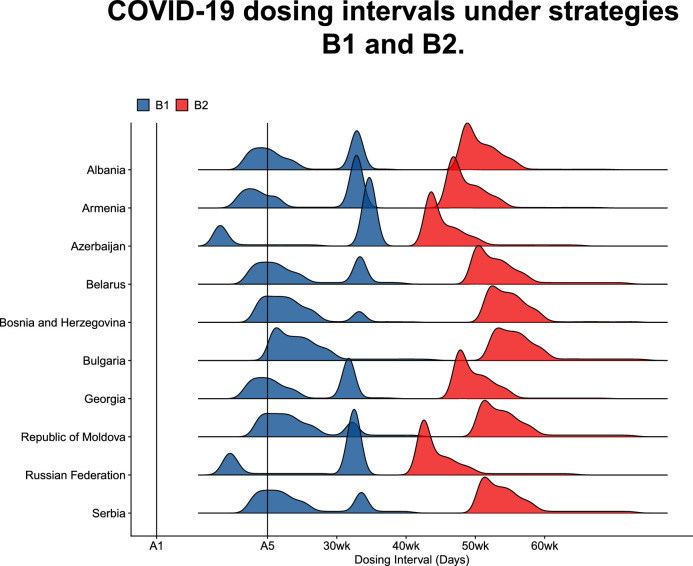
**COVID-19 dosing intervals under strategies B1 and B2.** These dosing strategies do not prescribe fixed dosing intervals. Vaccine allocations depend on whether or not coverage goals have been met in specific target groups. The distributions are outputs from dose allocation algorithms that capture such conditional relationships. Refer to Table 1 for descriptions of vaccination strategies.

With a 24-week delay in vaccine supply, under strategies A1 and A5, individuals could receive their second doses at the dosing interval prescribed (i.e. 4-20 weeks). Under strategies B1 and B2, the second doses are not provided based on a fixed dosing interval - but on conditions that coverage targets have been met in target populations. The mean dosing intervals under strategy B1 range between 24 and 29 weeks and the median between 22 and 34 weeks. The mean dosing intervals under strategy B2 range between 45 and 57 weeks and the median between 43 and 56 weeks (Figure 3). Under strategy B1, dosing intervals are bi-modally distributed, with the first peak representing the dosing intervals of older adults and the second peak representing the dosing intervals of young adults. The mean dosing interval among older adults under strategy B1 is comparable to strategy A5 in most countries and even strategy A4 in some countries (e.g. Azerbaijan and Turkey).

In the sensitivity analysis with supply delay of 12 weeks, the dosing intervals for older adults under B1 were significantly shortened; with a supply delay of 52 weeks, the dosing intervals for the entire population were longer than baseline (i.e. 24 weeks) (See Supplemental Figs. S2 and S3 and Supplemental Table S4 for more details). Due to population age structure, the relationship between vaccine supply and population-level dosing intervals under B1 and B2 are not linear.

### Optimal strategies under the base case scenario

At the beginning of the simulated vaccine roll-out processes (i.e. 01 March 2021), from model fitting, we estimated immunity levels in 13 MICs in the WHO European region to range from 5.89% to 30.8%.

**Figure 4:**
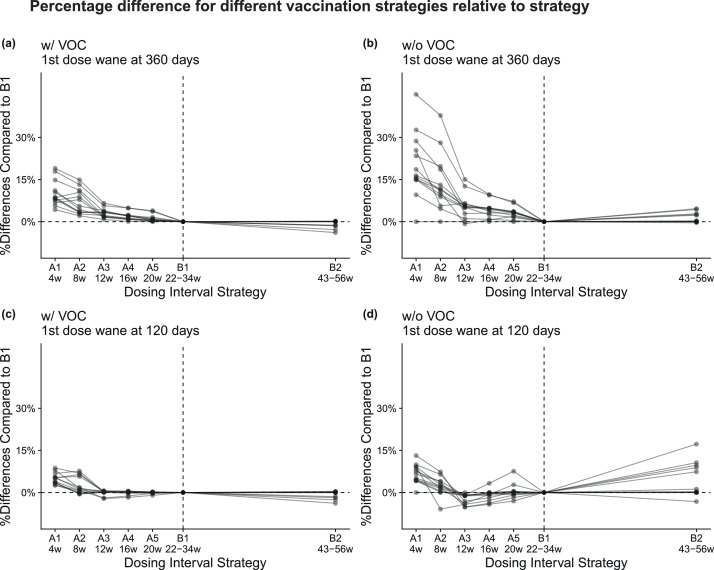
**Percentage difference in mortality for different vaccination strategies relative to strategy B1.** Each line represents a country. Detailed descriptions of the vaccine dosing strategies can be found in Table 1. Mean dosing intervals are presented as the second rows of x-axes labels (unit = weeks). Results from sensitivity analyses around the first dose waning durations (360 days vs 120 days) and the emergence of variants of concern (VOCs) are also presented.

Assuming VOC emergence and a mean first-dose waning duration of 360 days, B1 and B2 were optimal strategies for minimising COVID-19 mortality in all countries investigated and were comparable with each other (Figure 4 a). Strategy A1 may be associated with on average 10.1% higher cumulative mortality between 01 March 2021 and 31 December 2022 [range: 4.3% - 19.0%; n = 13 (countries)]. There is a negative association between dosing interval and relative cumulative mortality. Sensitivity analyses around vaccine supply delay show that under a severe delay (i.e. 52 weeks), A5 emerged as the optimal strategy for a small number of countries, although in these cases, the difference between A5, B1 and B2 remained small (i.e. within 5%) (see Supplemental Fig. S4).

Without VOC emergence, the advantages of strategy B1 are more evident (Figure 4 b). The negative association between dosing interval and COVID-19 mortality is also observed. However, strategy B2 is worse off than B1 in Albania, Azerbaijan, and Turkey by more than 2.5% (using the mortality under B1 as the denominator). Strategy A1 is associated with an average of 20.1% increase in cumulative mortality between 01 March 2021 and 31 December 2022 [range: 0%–45.4%; n = 13]. Note that in countries where no additional outbreak occurs during this period, the proportional difference between any strategies relative to strategy B1 is 0. The superiority of B1 is robust to changes in supply delay levels tested in the sensitivity analyses (see Supplemental Fig. S4).

Assuming a mean first-dose waning duration of 120 days, the superiority of strategy B1 is no longer evident. Optimal dosing strategies are shifted towards the range of A2 to A5 (8 to 20 weeks, Figure 4, c, d). We conducted additional sensitivity analyses using mean waning duration of 60 and 90 days and found that these parameters may further shift the optimal dosing intervals in some countries to as short as eight weeks (see Supplemental Fig. S5).

### Sensitivity analyses around vaccine efficacy

There is still considerable uncertainty around the relationship between dosing interval and the vaccine efficacy achievable after the second dose of the COVID-19 vaccines.^31^ In this study, we examined the extreme case where vaccine efficacy incrementally increases as dosing interval gets longer (Figure 1, b). We fixed the first dose infection-reducing vaccine efficacy at 65% to make it comparable to the results from the base case scenarios.

With VOC emergence, the comparative advantage of strategy B1 and B2 compared to strategies A1-A5 becomes slightly higher (Supplemental Fig. S6). Without VOC emergence, however, the advantage of strategy B1 and B2 over A1-A5 are of smaller magnitude if we consider the positive association between dosing interval and post-second-dose VEs.

While varying four dimensions of vaccine efficacy (i.e. first and second doses infection- and disease-reducing effects), we found that strategies B1 and B2 were comparable (within 5% difference) and advantageous for all countries investigated, consistent with the baseline results (Supplemental Fig. S7).

### Benefit-risk analysis

**Figure 5:**
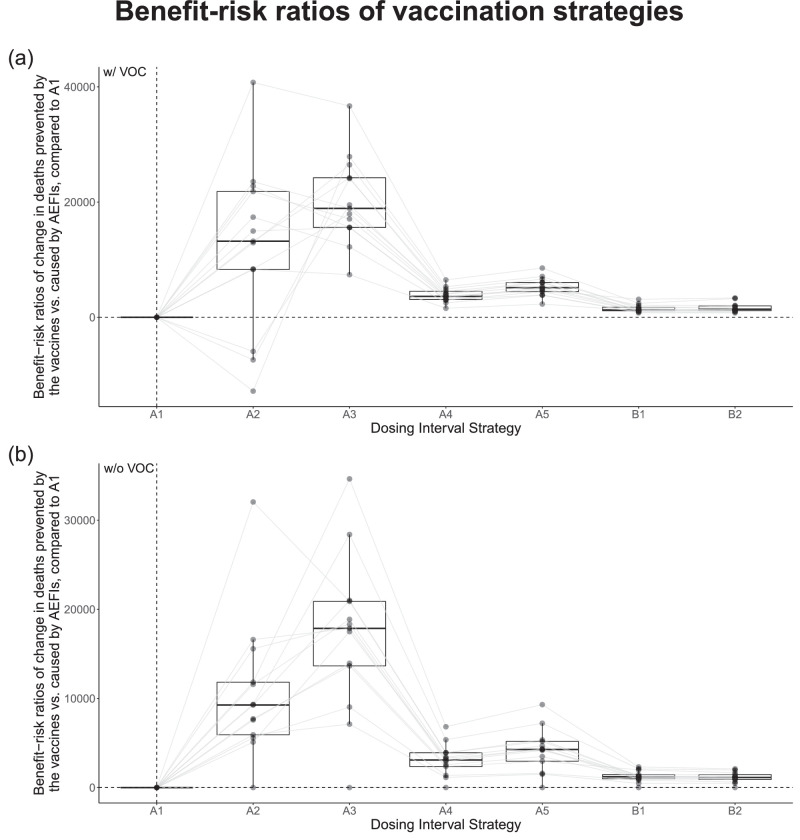
**Benefit-risk ratios of vaccination strategies.** Benefit-risk ratios of the different dosing interval strategies in comparison to strategy A1 are presented. Strategies A1-A5 and B1-B2 are arranged broadly based on mean effective dosing intervals. Each line represents one country. The benefit-risk ratios quantify the change in deaths prevented by the vaccines versus caused by AEFIs as compared to strategy A1. Refer to Table 1 for descriptions of vaccination strategies.

The additional deaths prevented by dosing strategies compared to the reference strategy A1 was always greater than the additional deaths due to fatal adverse events caused by these strategies. Most benefit-risk ratios of vaccination strategies in comparison to reference strategy A1 were greater than the additional harms (97.8%; n=178/182, with and without VOC combined); in four ratios, the benefits of strategy A2 or B2 were smaller than those of strategy A1 (A2 in Bosnia and Herzegovina, Serbia and Ukraine and B2 in Serbia). The additional benefits for strategies A2 and A3 were much greater than the additional harms among all other vaccination strategies, favouring a dosing interval of 8-12 weeks (compared to 4 weeks). The dominant benefit of strategy A3 was even more pronounced in the absence of VOC emergence (Figure 5 B).

Although we did not include the health and societal benefits of avoiding non-fatal SARS-CoV-2 positive cases, the general trend of additional population health outcomes (i.e., cases and severe disease, Supplemental Figs. S8 and S9) follows a similar trend as mortality, which indicates that including additional benefits should result in similar findings as shown in Figure 5.

## Discussion

We explored the health implications of different COVID-19 vaccine dosing interval scenarios in 13 middle-income countries in Europe. We explored seven strategies that involved either fixed dosing intervals of between 4 weeks and 20 weeks or a conditional policy that depended on vaccination goals being met. We found that vaccinating all older adults or adults with the first dose (strategies B1 and B2) resulted in the lowest COVID-19 cumulative mortality, while a dosing interval of 4 weeks (strategy A1) resulted in the largest number of cumulative mortality. Small cumulative mortality was associated with an early increase in the proportions of the population vaccinated with the first dose.

The optimal dosing strategies we have identified are all significantly longer than label recommendations (i.e. 3-4 weeks), which were based on clinical trial designs. In future vaccine development efforts during a pandemic, besides biological and logistic factors, implementation factors may also be factored in when designing dosing intervals for trials. Additional dosing schedules that allow for vaccine policy flexibility should be explored as soon as possible.

The results we have observed are robust to different vaccine characteristics assumptions of vaccine efficacy estimates versus dosing interval relationships and to different vaccine supply delays tested in this study. We conservatively assumed baseline vaccine characteristics based on AZD 1222. Using vaccine candidates with potentially slower first dose waning profile (e.g. mRNA vaccines),^32^ the advantage of strategies B1 and B2 will be even more evident.

Under the circulation of the wildtype and subsequently the Delta variant as modelled in this study, there is a negative association between dosing intervals and cumulative mortality when mean dosing intervals range between 4 and 26 weeks. This association may not persist with even longer dosing intervals of 43-56 weeks (as in strategy B2). While longer-than-currently recommended dosing intervals may be beneficial in reducing COVID-19 mortality, exceptionally long dosing intervals should be cautioned against. In this study, we tested a set of VOC characteristics approximating the Delta variant (by transmissibility and severity) assumed based on existing literature.^16^^,^^18^^,^^25^ There is additional uncertainty around these parameters that are not captured in our study. Such uncertainty and other characteristics of VOCs may alter our results. A faster waning of the protection derived by the first dose made strategies with shorter, fixed dosing intervals (i.e., A2-4) more favourable. A more transmissible yet less severe VOC (like Omicron) will likely shrink the relative difference between different dosing interval strategies – at which point, other factors (e.g., operational) should drive decisions.

When trading off the benefits of the vaccines in preventing deaths versus the harm from deaths caused by adverse events following immunisation, we found that the additional benefits of the strategies compared to strategy A1 exceeded the additional risks for most dosing interval scenarios. The ratio of benefits to risks was highest for strategies A2-A3 (intervals of 8-12 weeks), following the age-dependent rates of (prevented) deaths from COVID-19 and adverse events. Including additional outcomes and benefits for society is expected to lead to similar results.

Related studies in high-income country settings of Canada, Netherlands, the United Kingdom, and the United States also found that delaying the second dose of a two-dose COVID-19 vaccine series is beneficial.^8^^,^^33^, ^34^, ^35^ Moghadas et al. used an agent-based model to compare two strategies of either vaccinating more individuals with the first dose and delaying the second dose or administering the 2-dose series according to the recommended dose spacing for Pfizer-BioNTech (BNT162b2) and Moderna (mRNA-1273) vaccines.^8^ They suggested that depending on pre-existing immunity levels, additional hospitalisations and deaths could be averted by delaying the second dose due to vaccine prioritisation of individuals at higher risk of severe outcomes. Romero-Brufau et al. conducted a similar study to assess the cumulative public health impact over 6 months for delaying the second dose of Pfizer-BioNTech and Moderna vaccines and inferred that the delayed second-dose strategy for people under 65 years was a favourable strategy.^10^ Based on evidence synthesis of related studies^36^, ^37^, ^38^ and our study, we infer that for two-dose vaccines, the optimal dosing interval depends on multiple factors, including vaccine efficacy and effectiveness, waning dynamics of vaccine-induced immunity, vaccination coverage, vaccine supply rates, pre-existing naturally acquired immunity, and country-specific vaccine prioritisation plans.

In our study, we fitted a dynamic transmission model using reported daily COVID-19 mortality in 13 middle-income countries of Europe. We incorporated important epidemic dynamics, including VOC emergence and dosing dynamics (e.g. post-first-dose waning and the co-existence between natural infection and vaccination history). We explored a wide range of dosing interval strategies of prime interest to countries that may face constraints in vaccine supply. We performed extensive sensitivity analyses around key epidemiological parameters like the emergence of variants of concern and vaccine efficacy estimates. We extended the analysis by trading off the vaccines' benefits against the potential harm from adverse events following immunisation with the COVID-19 vaccines.

We captured between-country variability through population age structure, contact matrices, mobility and government response stringency index. However, the effect of these variables on SARS-CoV-2 transmission may differ between countries due to additional factors that we were not able to capture. For example, the Google mobility index we used to represent mobility may be less representative in countries with lower smartphone usage. The relationship between the mobility index and interpersonal contacts was established using data from the United Kingdom, which may be partially representative of other countries.

We could not extend the fitting window of this study further into 2021 or include spatial diffusion component (as did Cot et al.^39^) due to data availability issues (e.g. age-specific vaccine uptake, vaccine products in-use, strain-specific test positive rates, number of travellers between countries). Such data, have they become available, could improve model fit for future research. We have not incorporated reactive public health and social measures (e.g. lockdown enacted due to surging infections) as the specific implications are uncertain and country-specific. We were unable to capture breakthrough infections or vaccine waning among vaccinated individuals who have experienced previous infections. These pathways are biologically sound but would make it challenging to track vaccine allocations (e.g. making sure everyone only receives two doses). Given that these mechanisms are not widely characterised by empirical data, we did not include them in this study. We also did not explicitly look into the effect of vaccine reformulations with Omicron-specific strains that may become available later in 2022.

Our study shows that a dose-specific roll-out strategy that led to an average six-month dosing interval, which is substantially longer than the current label recommendation for most vaccine products available in the European market, may be able to minimise COVID-19 mortality in the MICs in Europe. Countries included in this study have diverse population age structures, contact patterns, and epidemic histories – the overall conclusions are valuable to COVID-19 vaccine dosing policy-making in LMICs elsewhere in the world.

## Contributors

YL, FGS, MJ, and KA conceptualised the study. YL, CABP, and FGS developed the model and conducted the health impact assessment and benefit-risk analysis. RCB compiled the evidence on vaccine efficacy estimates. JHK and SF interpreted the comparative analysis of dosing interval strategies. YL wrote the original draft, and all authors contributed to reviewing and editing the manuscript for important intellectual content and have approved the final version.

## Declaration of Interests

We declare no competing interests.

## Appendix Supplementary materials

Image, application 1

Image, application 2
