# Supplementary material for: Dosing interval strategies for two-dose COVID-19 vaccination in 13 middle-income countries of Europe: Health impact modelling and benefit-risk analysis
Source: Lancet Reg Health Eur. 2022 Apr 11;17:100381. doi: 10.1016/j.lanepe.2022.100381 (PMC8996067; doi:10.1016/j.lanepe.2022.100381)
Supplement: Supplementary file 1 [file mmc1.docx]

**[Supplemental Material]**

**Dosing interval strategies for two-dose COVID-19 vaccination in 13 middle-income countries of Europe: health impact modelling and benefit-risk analysis**

Yang Liu,^1,2 #^ Carl AB Pearson,^1,2^ Frank G Sandmann,^1,2,3 *^ Rosanna C Barnard,^1,2^ Jong-Hoon Kim,^4^ CMMID COVID-19 Working Group, Stefan Flasche,^1,2^ Mark Jit,^1,2.3^ Kaja Abbas^1,2^

^1^ Centre for Mathematical Modelling of Infectious Diseases, London School of Hygiene & Tropical Medicine, London, United Kingdom

^2^ Department of Infectious Disease Epidemiology, Faculty of Epidemiology and Population Health, London School of Hygiene & Tropical Medicine, London, United Kingdom

^3^ Statistics, Modelling and Economics Department, National Infection Service, UK Health Security Agency (UK HSA), London, United Kingdom

^4^ International Vaccine Institute, Seoul, South Korea

* Current address: European Centre for Disease Prevention and Control (ECDC)

Contents

[Supplemental Tables 3](#_Toc95758155)

[Table S1. Model Parameters 3](#_Toc95758156)

[Table S2. Model Description 5](#_Toc95758157)

[Table S3. The rationale behind vaccine effectiveness estimates 8](#_Toc95758158)

[Table S4. Summary statistics of effective dosing intervals under B1 and B2 given different vaccine supply delays 10](#_Toc95758159)

[Supplemental Figures 11](#_Toc95758160)

[Figure S1. Vaccine roll-out progress among LMICs of the WHO European Regions (n = 20) 11](#_Toc95758161)

[Figure S2. Effective dosing interval given 12 weeks of supply delay under strategies B1 and B2 12](#_Toc95758162)

[Figure S3. Effective dosing interval given 52 weeks of supply delay under strategies B1 and B2 13](#_Toc95758163)

[Figure S4. Relative performance of dosing interval strategies given alternative supply delay levels 14](#_Toc95758164)

[Figure S5. Sensitivity analysis using different waning duration (60 and 90 days) by country 15](#_Toc95758165)

[Figure S6. Outcomes using a dynamic relationship between dosing interval and the vaccine efficacy achievable after both doses. 16](#_Toc95758166)

[Figure S7. Sensitivity analyses around four dimensions of vaccine efficacy (infection- and disease-reducing vaccine efficacy after first and second doses). 17](#_Toc95758167)

[Figure S8. Different outcomes by dosing strategy while accounting for VOC emergence 19](#_Toc95758168)

[Figure S9. Different outcomes by dosing strategy without accounting for VOC emergence 20](#_Toc95758169)

[Supplemental Methods 21](#_Toc95758170)

[Population contact patterns 21](#_Toc95758171)

[Calculating COVID-19 mortality and hospitalisation 22](#_Toc95758172)

[Setting up the sensitivity analyses around the vaccine efficacies 23](#_Toc95758173)

[TREND checklist 24](#_Toc95758174)

##

# Supplemental Tables

### Table S1. Model Parameters

| **Disease process characteristics** | | |
| --- | --- | --- |
| **Parameter** | **Value** | **Source** |
| Age-specific susceptibility ($u_{i}$) | 0.38 - 0.88 | Davies et al.[(1)](https://sciwheel.com/work/citation?ids=9102939&pre=&suf=&sa=0) |
| Age-specific clinical progression rates ($y_{i}$) | 0.21 - 0.70 | Davies et al[(1)](https://sciwheel.com/work/citation?ids=9102939&pre=&suf=&sa=0) |
| Age-specific infection fatality rates | Raw input: 5.2e-6 - 0.13  By age group: 6.7e-6 – 8.1e-2 | Levin et al.[(2)](https://sciwheel.com/work/citation?ids=10252041&pre=&suf=&sa=0) |
| Age- and country-specific within-population contact pattern  ($C_{i,j,0}$) | Country-specific | Prem et al.[(3)](https://sciwheel.com/work/citation?ids=11703857&pre=&suf=&sa=0) |
| Country-specific population age structures | Country-specific | United Nations[(4)](https://sciwheel.com/work/citation?ids=8436701&pre=&suf=&sa=0&dbf=0) |
| Relationship between mobility and population contact pattern | Defined by linear and nonlinear functions for the *workplace* and *other* settings, respectively. | Davies et al. by fitting to UK data[(5)](https://sciwheel.com/work/citation?ids=10245997&pre=&suf=&sa=0) |
| Latent period ($1/\sigma$) | ~gamma (μ = 2.5, k = 2.5) | Pearson et al.[(6)](https://sciwheel.com/work/citation?ids=12468223&pre=&suf=&sa=0&dbf=0)  Davies et al.[(7)](https://sciwheel.com/work/citation?ids=9011259&pre=&suf=&sa=0)  Davies et al.[(5)](https://sciwheel.com/work/citation?ids=10245997&pre=&suf=&sa=0)  Bi et al.[(8)](https://sciwheel.com/work/citation?ids=8792615&pre=&suf=&sa=0)  Liu et al.[(9)](https://sciwheel.com/work/citation?ids=8738168&pre=&suf=&sa=0)  Linton et al.[(10)](https://sciwheel.com/work/citation?ids=8415907&pre=&suf=&sa=0)  Nishiura et al.[(11)](https://sciwheel.com/work/citation?ids=8415585&pre=&suf=&sa=0) |
| Duration of preclinical infectiousness ($1/\gamma_{p}$) | ~gamma (μ = 1.5, k = 4) |
| Duration of clinical infectiousness ($1/\gamma_{c}$) | ~gamma (μ = 3.5, k = 4) |
| Duration of subclinical infectiousness ($1/\gamma_{s}$) | ~gamma (μ = 5, k = 4) | Assumed, consistent with Davies et al.[(7)](https://sciwheel.com/work/citation?ids=9011259&pre=&suf=&sa=0) |
| Relative infectiousness of subclinical infections compared to clinical infections ($f$) | 0.5 | Assumed, consistent with Davies et al.[(7)](https://sciwheel.com/work/citation?ids=9011259&pre=&suf=&sa=0) |
| Duration of infection-induced immunity  (1/$\omega_{n}$) | 3 years | Hall et al.[(12)](https://sciwheel.com/work/citation?ids=10879406&pre=&suf=&sa=0) |
|  | | |
| **Mortality, mobility, and non-pharmaceutical intervention characteristics** | | |
| **Parameter** | | **Source** |
| Country-level daily COVID-19 mortality (including 7-day rolling average) | | Ritchie et al.[(13)](https://sciwheel.com/work/citation?ids=11703906&pre=&suf=&sa=0) |
| Observed country-specific community mobility | | Google[(14)](https://sciwheel.com/work/citation?ids=11703648&pre=&suf=&sa=0) |
| COVID-19 Government Response Stringency Index and Government Response Tracker by country | | Hale et al.[(15)](https://sciwheel.com/work/citation?ids=10678231&pre=&suf=&sa=0) |
| **Vaccine characteristics** | | |
| **Parameter** | **Value** | **Source** |
| First dose protection duration (1/$\omega_{v}$) | Baseline = 360 days  Sensitivity analysis: 120 days | Voysey et al.[(16)](https://sciwheel.com/work/citation?ids=10525817&pre=&suf=&sa=0) |
| Vaccine roll-out scenarios | 0.03 by mid-2021 and 0.2 by end of 2021, 0.5 by end of 2022 | Gavi, the vaccine alliance[(17)](https://sciwheel.com/work/citation?ids=11703883&pre=&suf=&sa=0)  World Health Organisation[(18,19)](https://sciwheel.com/work/citation?ids=11703893,11703889&pre=&pre=&suf=&suf=&sa=0,0) |
| Maximum willingness to receive vaccination | 0.7 for those between 20-59 and 0.9 for those above 60 | Wouter et al.[(20)](https://sciwheel.com/work/citation?ids=10480992&pre=&suf=&sa=0)  Robinson et al.[(21)](https://sciwheel.com/work/citation?ids=11230457&pre=&suf=&sa=0)  UK Government[(22)](https://sciwheel.com/work/citation?ids=11703899&pre=&suf=&sa=0) |
| **Variant of Concern characteristics** | | |
| **Parameter** | **Value** | **Source** |
| Increase in transmissibility^ | 50% | Barnard et al. [(23)](https://sciwheel.com/work/citation?ids=11702792&pre=&suf=&sa=0&dbf=0)  Davies et al.  [(24)](https://sciwheel.com/work/citation?ids=10593953&pre=&suf=&sa=0&dbf=0) |
| Introduction time^ | 15 April 2021 | Mishra et al. [(25)](https://sciwheel.com/work/citation?ids=11695059&pre=&suf=&sa=0&dbf=0) |

^ The increase in transmissibility is a non-strain specific relative measure that compares post introduction level to pre introduction level. We used the introduction time of the Delta variant to the UK to approximate the introduction time for countries we are studying here, where variants surveillance may be limited (see <https://worldhealthorg.shinyapps.io/euro-covid19/>). The implication is that before the introduction time, wildetype and alpha variants were circulating with different mixture; after the introduction time, alpha and delta variants were circulating with different mixture. The 50% increase in transmissibility is an approximation based on the relative difference between the Delta and Alpha variants and between Alpha and wildtype.[(23,24)](https://sciwheel.com/work/citation?ids=10593953,11702792&pre=&pre=&suf=&suf=&sa=0,0&dbf=0&dbf=0)

### Table S2. Model Description

| $S_{i}(t)$ | Susceptible individuals among age group $i$ at time $t$ |  |
| --- | --- | --- |
|  | $S_{i}(t + 1)=S_{i}(t)\cdot{(1-\lambda}_{i}(t)) - v_{1i}(t+1)\cdot\frac{S_{i}(t)}{P_{1i}(t)} +R_{i}(t)\cdot\omega_{n}$ | [1] |
| $V_{1i}(t)$ | Individuals among age group $i$ who received their first doses at time $t$ |  |
|  | $V_{1i}(t+1)={V_{1i}(t)\cdot(1-\lambda_{i}(t)\cdot(1-{ve}_{i}) -\omega_{v}) + v}_{1i}(t+1)\cdot\frac{S_{i}(t)}{P_{1i}(t)}-$ $v_{2i}(t+1)\cdot\frac{V_{1i}(t)}{P_{2i}(t)}$ | [2] |
| $S_{wi}(t)$ | Individuals among age group $i$ who received their first doses but the protection has waned at time $t$ |  |
|  | $S_{wi}\left( t+1 \right)={S_{wi}(t)\cdot{(1-\lambda}_{i}(t))+V}_{1i}\left( t \right)\cdot\omega_{v}- v_{2i}(t+1)\cdot\frac{S_{wi}(t)}{P_{2i}(t)}$ | [3] |
| $V_{2i}(t)$ | Individuals among age group $i$ who received their second doses at time $t$ |  |
|  | $V_{2i}\left( t+1 \right)=V_{2i}\left( t \right)\cdot(1- \lambda_{i}\left( t \right)\cdot\left( 1-{v2e}_{i} \right))+$ $v_{2i}\left( t+1 \right)\cdot(\frac{S_{wi}\left( t \right)}{P_{2i}\left( t \right)} + \frac{V_{1i}\left( t \right)}{P_{2i}\left( t \right)})$ | [4] |
| $E_{i}(t)$ | Exposed individuals who are not protected by vaccines (either unvaccinated or have their first doses already waned) among age group $i$ at time $t$ |  |
|  | $E_{i}\left( t+1 \right)=E_{i}\left( t \right)\cdot(1- \sigma)+S_{i}(t)\cdot\lambda_{i}(t)+S_{wi}(t)\cdot\lambda_{i}(t)$ | [5] |
| $E_{vi}(t)$ | Exposed individuals who are protected by one dose of the vaccine among age group $i$ at time $t$ |  |
|  | $E_{vi}\left( t+1 \right)=E_{vi}\left( t \right)\cdot\left( 1-\sigma\right)+V_{1i}\left( t \right)\cdot\lambda_{i}\left( t \right)\cdot\left( 1-{ve}_{i} \right)$ | [6] |
| $E_{v2i}(t)$ | Exposed individuals who are protected by one dose of the vaccine among age group $i$ at time $t$ |  |
|  | $E_{v2i}(t+1)={E_{v2i}\left( t \right)\cdot\left( 1-\sigma\right)+V}_{2i}(t)\cdot\lambda_{i}(t)\cdot(1-{v2e}_{i})$ | [7] |
| $I_{pi}(t)$ | Pre-clinical infectious individuals among age group $i$ at time $t$ |  |
|  | $I_{pi}(t+1)=I_{pi}(t)\cdot{(1-\gamma}_{p})+E_{i}(t) \cdot\sigma\cdot y_{i} +$ $E_{vi}(t) \cdot\sigma\cdot y_{i} \cdot(1-{ve}_{d}) + E_{v2i}(t) \cdot\sigma\cdot y_{i} \cdot(1-{v2e}_{d})$ | [8] |
| $I_{ci}(t)$ | Clinical infectious individuals among age group $i$ at time $t$ |  |
|  | $I_{ci}(t+1)=I_{ci}(t) \cdot\left( 1-\gamma_{c} \right)+I_{pi}(t)\cdot\gamma_{p}$ | [9] |
| $I_{si}(t)$ | Subclinical infectious individuals among age group $i$ at time $t$ |  |
|  | $I_{si}(t+1)=I_{si}(t)\cdot{(1-\gamma}_{s})+ E_{i}(t) \cdot\sigma\cdot{(1-y}_{i}) +$ $E_{vi}(t) \cdot\sigma\cdot{(1 - y}_{i} \cdot(1-{ve}_{d})) +$ $E_{v2i}(t) \cdot\sigma\cdot{(1 - y}_{i} \cdot(1-{v2e}_{d}))$ | [10] |
| $R_{i}(t)$ | Recovered individuals |  |
|  | $R_{i}\left( t+1 \right)=R_{i}\left( t \right)\cdot\left( 1-\omega_{n} \right)+ I_{si}(t)\cdot\gamma_{s} +I_{ci}(t) \cdot\gamma_{c} -$ $v_{1i}(t+1)\cdot\frac{R_{i}(t)}{P_{1i}}$ | [11] |
| $R_{vi}(t)$ | Individuals who have recovered from their previous infections and have received one dose |  |
|  | $R_{vi}\left( t+1 \right)=R_{vi}\left( t \right)+v_{1i}(t+1)\cdot\frac{R_{i}(t)}{P_{1i}}-v_{2i}(t)\cdot\frac{R_{vi}(t)}{P_{2i}(t)}$ | [12] |
| $R_{v2i}(t)$ | Individuals who have recovered from their previous infections and have received two doses |  |
|  | $R_{v2i}(t+1)=R_{v2i}\left( t \right)+v_{2i}(t)\cdot\frac{R_{v2i}(t)}{P_{2i}(t)}$ | [13] |

In which:

| $\lambda_{i}(t)$ | Is the force of infection on population $i$ at time $t$: $\lambda_{i}(t) = u_{i}\cdot\sum_{j = 1}^{J} C_{i,j,t}\cdot\frac{(I_{pj}(t) + I_{cj}(t) + I_{sj}(t)\cdot f)}{N_{j}}$ Where $j$ depicts age group, $J$ is 16, $f$ is the relative infectiousness of subclinical individuals compared to pre-clinical and clinical individuals (i.e. 50%), and $u_{i}$ is susceptibility. |
| --- | --- |
| $P_{1i}(t)$ | Is the population eligible for the first doses in the population $i$ at time $t$: $P_{1i}(t) = N_{i} -(V_{1i}(t) + S_{wi}(t) + R_{vi}(t) + E_{vi}(t)) -{(R}_{v2i}(t)+V_{2i}(t))$ |
| $P_{2i}(t)$ | Is the population eligible for the second doses in the population $i$ at time $t$: $P_{2i}(t) =V_{1i}(t) + S_{wi}(t) + R_{vi}(t) + E_{vi}(t)$ |
| $v_{1i}(t)$ | Is the number of doses to vaccinate the population with dose 1, this is pre-calculated based on vaccine dosing interval strategies. |
| $v_{2i}(t)$ | Is the number of doses to vaccinate the population with dose 2, this is pre-calculated based on vaccine dosing interval strategies. |

##

### Table S3. The rationale behind vaccine effectiveness estimates

Vaccine effectiveness against pre-B.1.1.7 and B.1.1.7 relevant evidence (from roadmap report prior to step 4). This table is originally prepared for a report prepared by Barnard et al.[(23)](https://sciwheel.com/work/citation?ids=11702792&pre=&suf=&sa=0)

| **Description** | **Relevant evidence, assumed value shown in bold** |
| --- | --- |
| Overall protection against infection for AstraZeneca dose 1 | Shrotri et al.[(26)](https://sciwheel.com/work/citation?ids=11407622&pre=&suf=&sa=0&dbf=0) results, secondary analyses, paragraph 1, p.8 adjusted hazard ratio 0.33 (0.16, 0.68) at 28-34 days post vaccination. Pritchard et al.[(27)](https://sciwheel.com/work/citation?ids=11163054&pre=&suf=&sa=0&dbf=0) supplementary Table 7, adjusted odds ratio >=21 days after first dose of AZ 0.36 (0.3, 0.45).  **0.67 (+28 days)** |
| Overall protection against disease for AstraZeneca dose 1 | Lopez Bernal et al.[(28)](https://sciwheel.com/work/citation?ids=11033114&pre=&suf=&sa=0) Table 3, ChAdOx1 adjusted odds ratio d1:28-34 0.4 (0.27-0.59), adjusting 0.6 up to equivalent estimate for protection against infection (see cell above)  **0.67 (+28 days) as for infection** |
| Overall protection against hospitalisation for AstraZeneca dose 1 | Vasileiou et al.[(29)](https://sciwheel.com/work/citation?ids=10948526&pre=&suf=&sa=0) Table 2, vaccine programme effect for ChAdOx1 21-27 days post first vaccine is 81% (72 to 87%), 28-34 days post first vaccine is 88% (75-94%), 35-41 days post first vaccine is 97% (63-100%). Smaller numbers. Table 3 splits analysis into age groups for ChAdOx1: 65-79 years 21-27 days post first dose 68% (31 to 85%), 80+ years 21-27 days post first dose 77% (63 to 86%) and 28-34 days post first dose 81% (60 to 91%). Small numbers for 65-79 years old and for 18-64 years old, so difficult to directly compare but overall the vaccine effect appears stronger in the younger (65-79 years) cohort than the older (80+) cohort, for the first three time points which enable comparison. Effect reversed for fourth time point. Ismail et al.[(30)](https://sciwheel.com/work/citation?ids=11708384&pre=&suf=&sa=0) estimate vaccine effectiveness against hospitalisation of 73% (60-81%) for 80+ year olds and 84% (74-89%) for 70-79 year olds, 28 days following the first dose of AZ. When analysis is not split across vaccine products, the same study estimates efficacy against hospitalisation of 80% (74-85%) for 80+ year olds and 82% (75-87%) for 70-79 year olds.  **0.845 (+28 days)** |
| Overall protection against mortality for AstraZeneca dose 1 | Lopez Bernal et al.[(31)](https://sciwheel.com/work/citation?ids=11120613&pre=&suf=&sa=0) estimated a hazard ratio of 0.45 (0.34 - 0.59) for cases vaccinated with one dose of AZ compared to unvaccinated cases, indicating an additional 55% (41-66%) protection against death given becoming a case for individuals vaccinated with one dose of AZ. Using the aforementioned estimate of a 55% increase and assuming this in addition to protection against disease of **0.67,** we get overall protection against mortality of 0.8515  **0.845 (+28 days)** |
| Overall protection against onward transmission for AstraZeneca dose 1 | Harris et al.[(32)](https://sciwheel.com/work/citation?ids=11708395&pre=&suf=&sa=0) estimate an odds ratio of infection for contacts of index cases vaccinated with ChAdOx1 (AstraZeneca) matched to contacts of unvaccinated index cases of 0.62 (95% 0.48-0.79), where the vaccinated index cases received their vaccine dose at least 21 days before testing positive.  **0.38 (+28 days)** |
| Overall protection against infection for AstraZeneca dose 2 | Shrotri et al.[(26)](https://sciwheel.com/work/citation?ids=11407622&pre=&suf=&sa=0&dbf=0) results, secondary analyses, paragraph 1, p.8 adjusted hazard ratio 0.32 (0.15, 0.66) at 35-48 days post vaccination  **0.68 (+14 days)** |
| Overall protection against disease for AstraZeneca dose 2 | Voysey et al.[(33)](https://sciwheel.com/work/citation?ids=10138638&pre=&suf=&sa=0) A randomised controlled trial for ChAdOx1 nCoV-19 vaccine AZD1222, Table 3, average of efficacies more than 14 days after a second dose for LD/SD and SD/SD in ‘COV002 (UK), age 18–55 years with >8 weeks’ interval between vaccine doses*’ row -> 0.778 = (0.9+0.656)/2  **0.78 (+14 days)** |
| Overall protection against hospitalisation for AstraZeneca dose 2 | Ismail et al.[(30)](https://sciwheel.com/work/citation?ids=11708384&pre=&suf=&sa=0) estimate vaccine effectiveness against hospitalisation of 92% (87-95%) 14 days after a second dose across both AZ and Pfizer vaccines  **0.9 (+14 days)** |
| Overall protection against mortality for AstraZeneca dose 2 | **0.95 (+14 days)** |
| Overall protection against onward transmission for AstraZeneca dose 2 | **0.5 (+14 days)** |

### Table S4. Summary statistics of effective dosing intervals under B1 and B2 given different vaccine supply delays

| Supply Delay | Strategy | Min | Median | Mean | Max |
| --- | --- | --- | --- | --- | --- |
| **24** | **B1** | **12.71** | **24.43** | **25.79** | **51.43** |
|  | **B2** | **41.85** | **50.29** | **50.52** | **75** |
| 12 | B1 | 9.57 | 32.14 | 25.84 | 49.71 |
|  | B2 | 41.14 | 49.14 | 48.31 | 69 |
| 52 | B1 | 16.14 | 31.29 | 30.69 | 52.71 |
|  | B2 | 52.42 | 66.43 | 66.14 | 89.29 |

# Supplemental Figures

### Figure S1. Vaccine roll-out progress among LMICs of the WHO European Regions (n = 20)


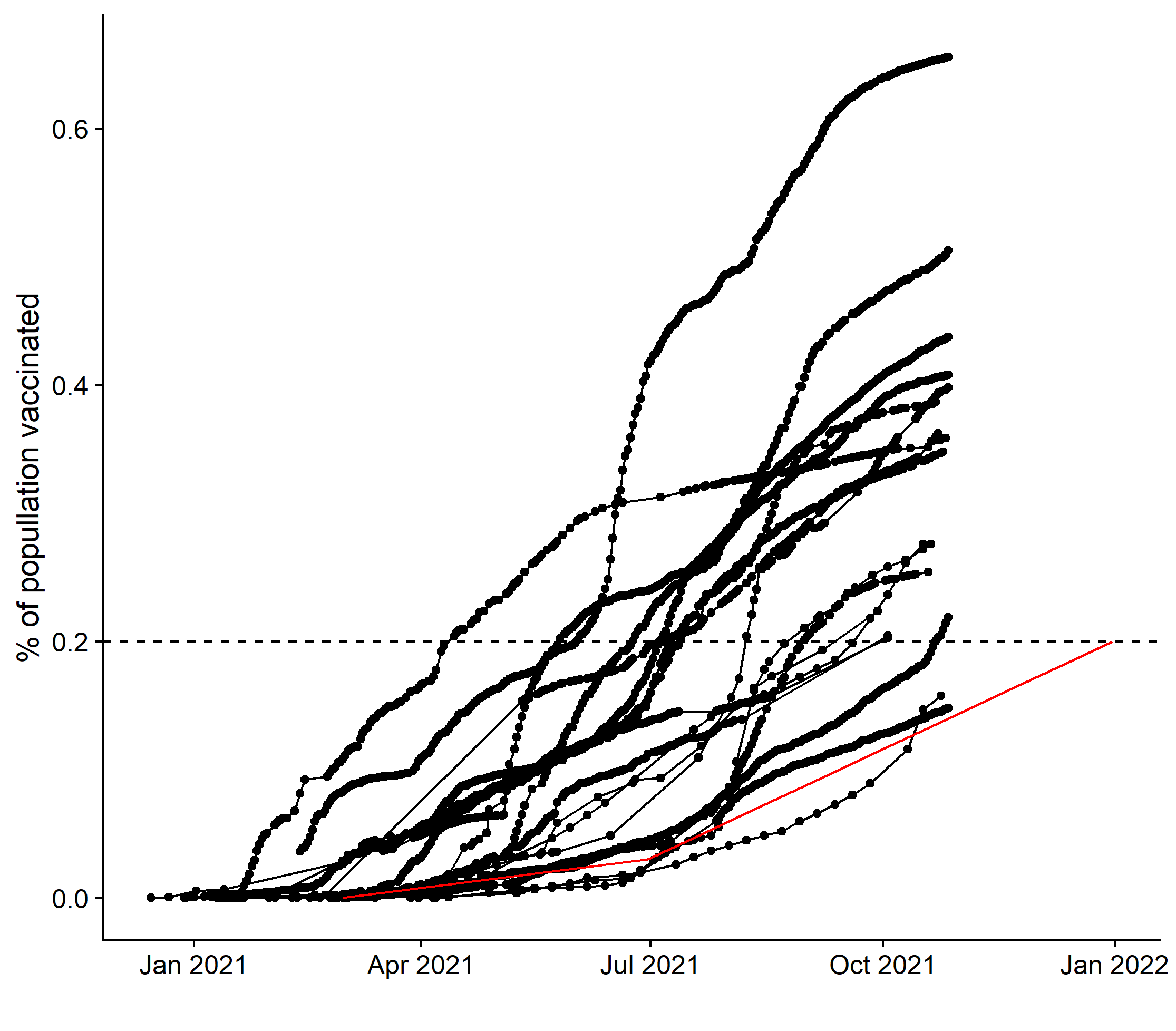


Caption: Each black dotted line represents a country; the red line represents the vaccine rollout strategy investigated in this study. Compared to the rest of the world, LMICs within the WHO European Region are relatively well off. We chose the slowest adaptor within the Region to resemble the slow vaccine row-out conditions that LMICs elsewhere in the world may face.

### Figure S2. Effective dosing interval given 12 weeks of supply delay under strategies B1 and B2


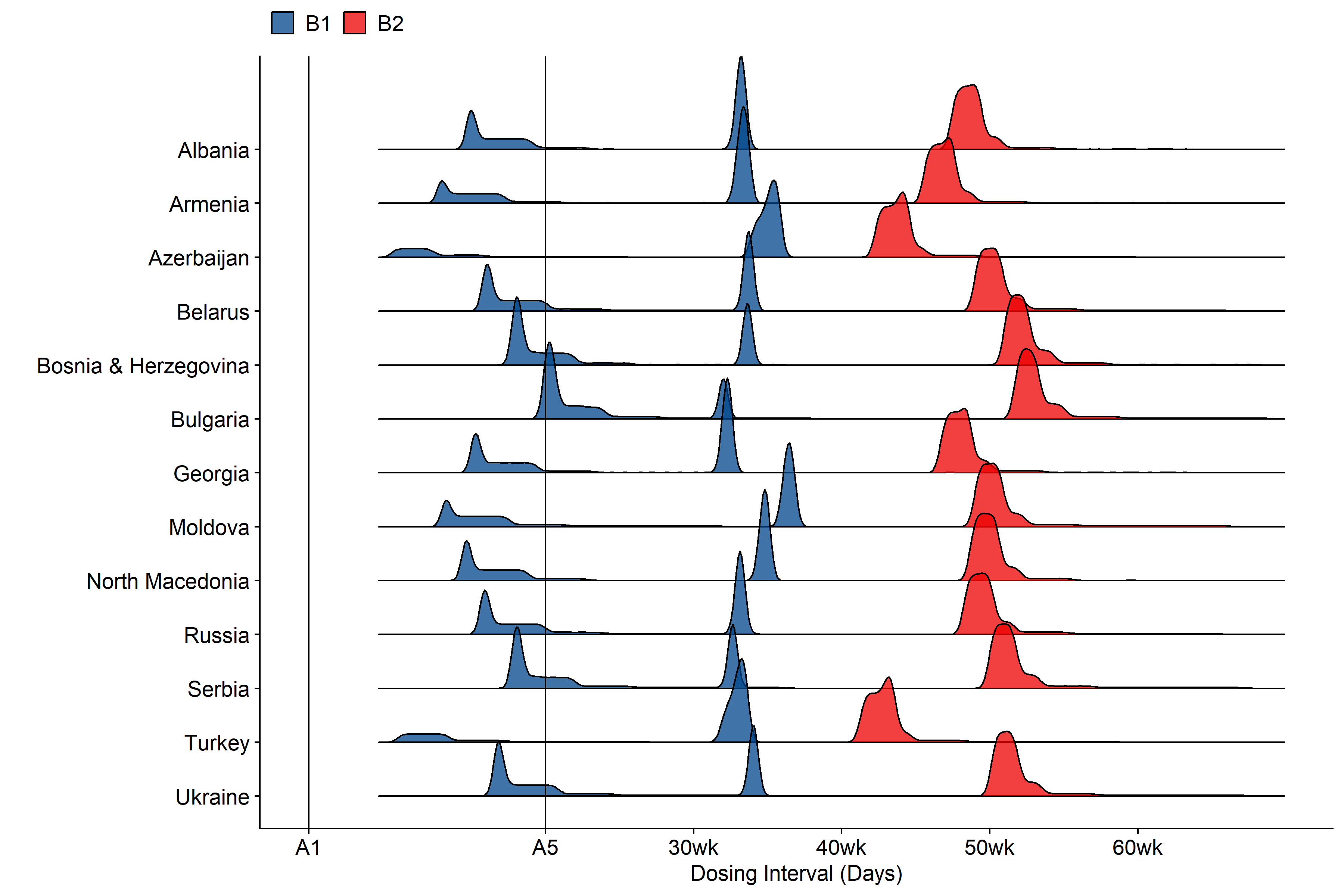


These dosing strategies do not prescribe fixed dosing intervals. Vaccine allocations depend on whether coverage goals have been met in certain target groups. The distributions are outputs from dose allocation algorithms that capture such conditional relationships. Refer to Table 1 in the main text for descriptions of vaccination strategies.

### Figure S3. Effective dosing interval given 52 weeks of supply delay under strategies B1 and B2


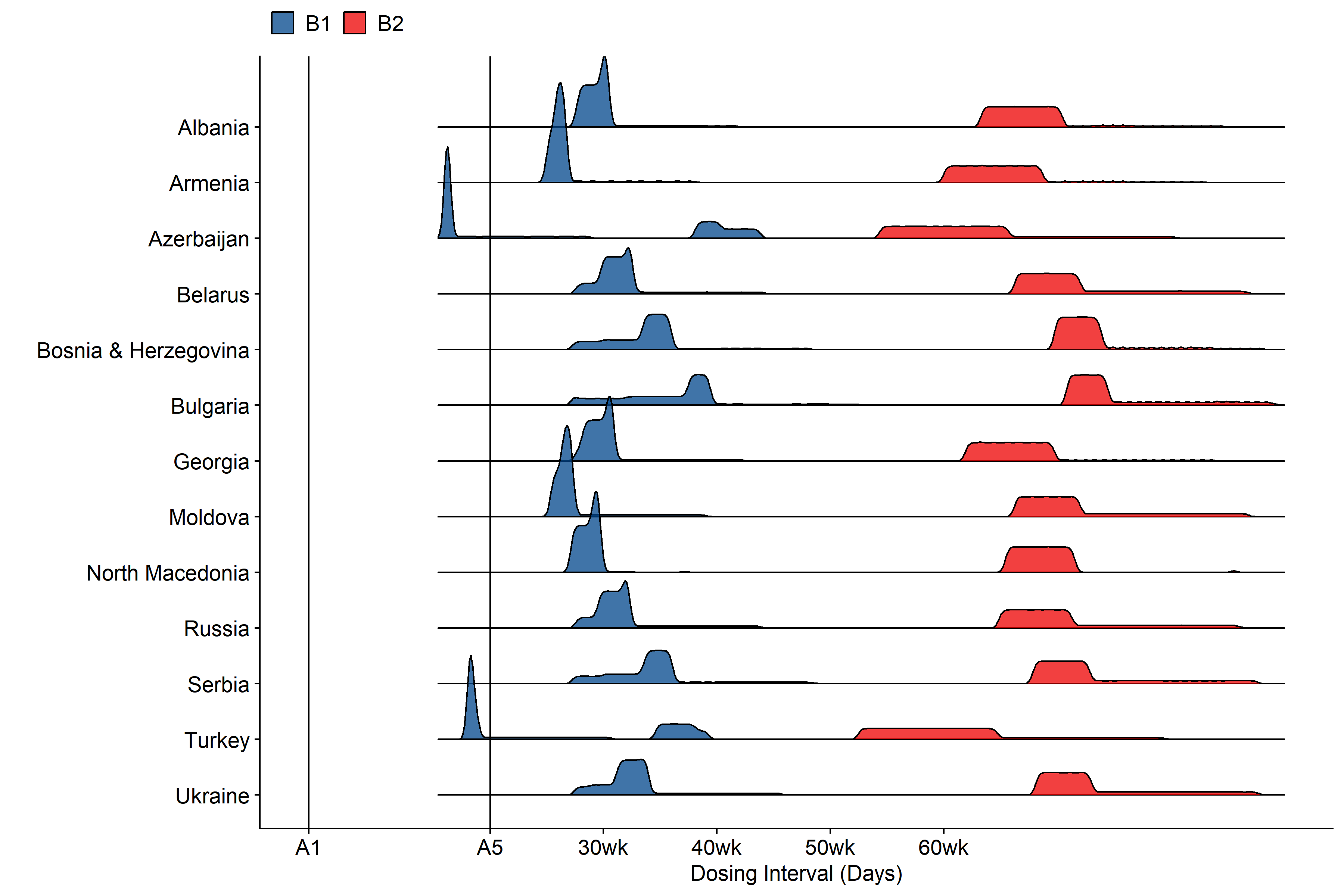


These dosing strategies do not prescribe fixed dosing intervals. Vaccine allocations depend on whether coverage goals have been met in certain target groups. The distributions are outputs from dose allocation algorithms that capture such conditional relationships. Refer to Table 1 in the main text for descriptions of vaccination strategies.

### Figure S4. Relative performance of dosing interval strategies given alternative supply delay levels


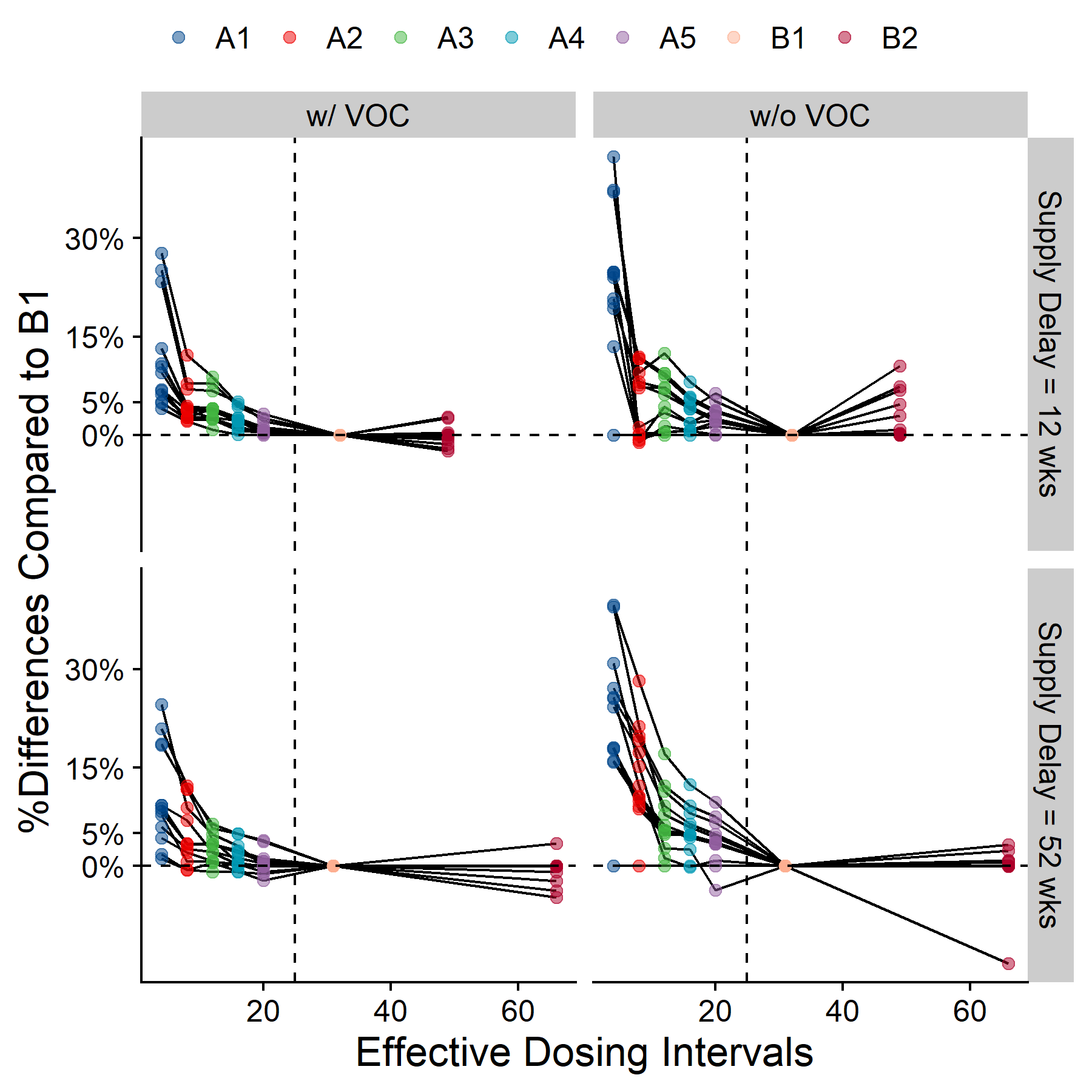


Each line represents a country. Detailed descriptions of the vaccine dosing strategies can be found in Table 1 in the main text. In this sensitivity analysis, we used the baseline waning duration of first dose (i.e. 360 days).

###
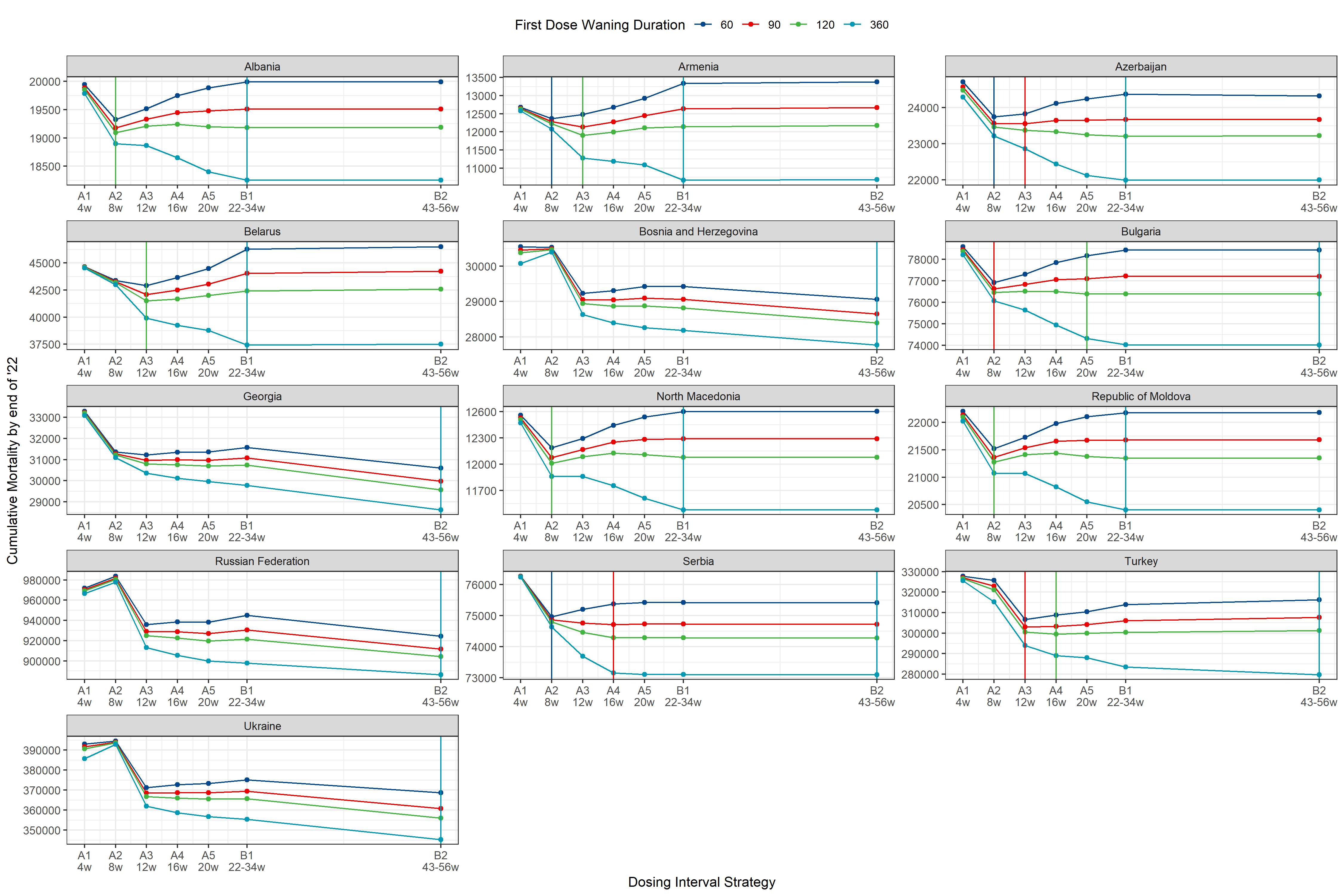
Figure S5. Sensitivity analysis using different waning duration (60 and 90 days) by country

The location (along the x-axis) indicates the optimal strategy which lead to the smallest number of cumulative mortalities; the colour of the vertical lines indicates the underlying waning duration assumed. We generally expect teal -> green -> red -> navy to move from right to left, incrementally. While this is true for nine of 13 countries, four countries’ optimal strategy was insensitive to the assumption around waning duration: Bosnia and Herzegovina, Georgia, Russian Federation and Ukraine. This is likely the result of a combination of factors including the population age structure and existing sizes of epidemics.

### Figure S6. Outcomes using a dynamic relationship between dosing interval and the vaccine efficacy achievable after both doses.

**
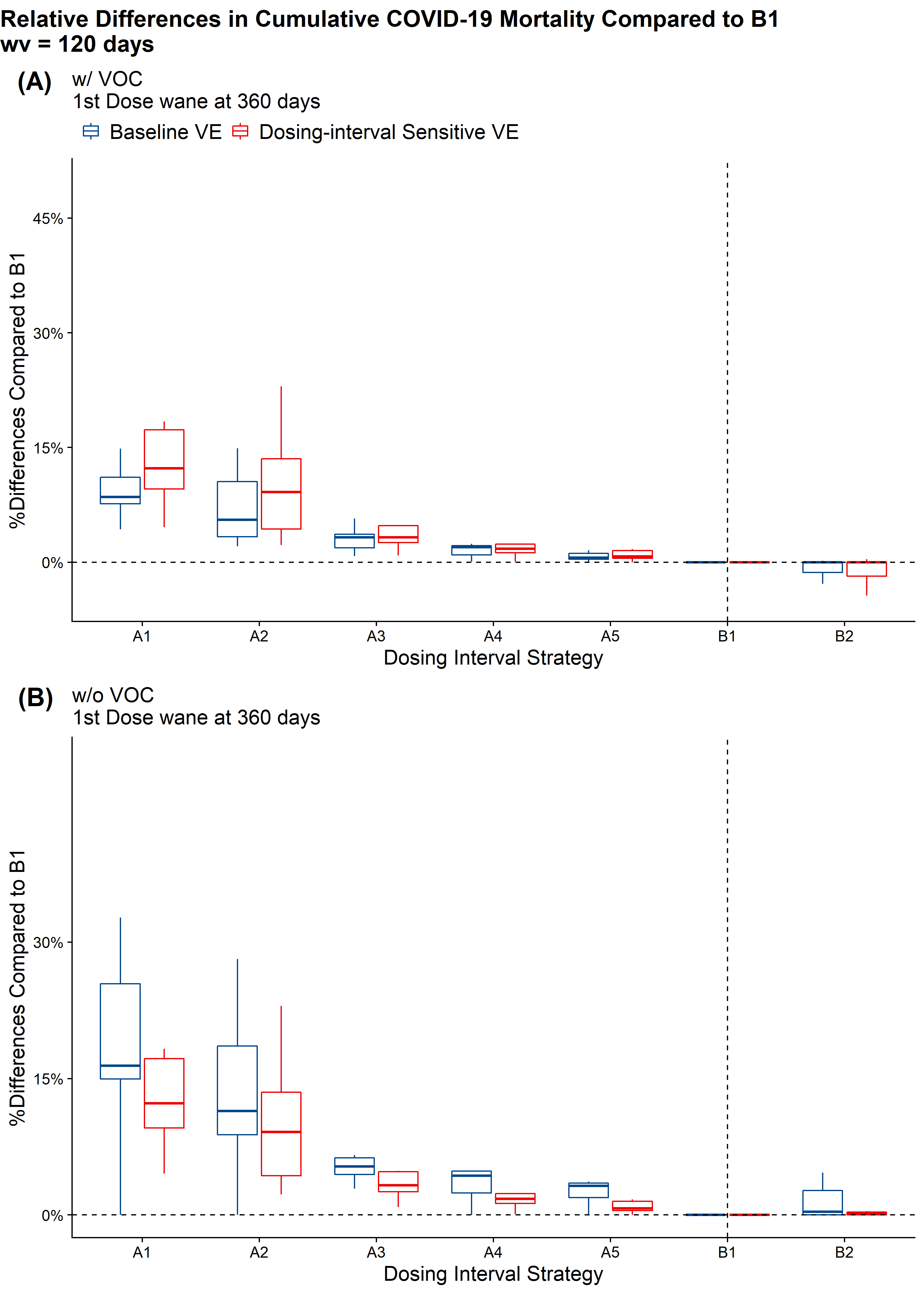
**

### Figure S7. Sensitivity analyses around four dimensions of vaccine efficacy (infection- and disease-reducing vaccine efficacy after first and second doses).

The black dashed line shows 0% difference, the set of orange dashed lines show +/- 1% differences, and the set of red dashed lines show +/- 5% differences. All percentage differences were calculated using the COVID-19 mortality under strategy B1.

**
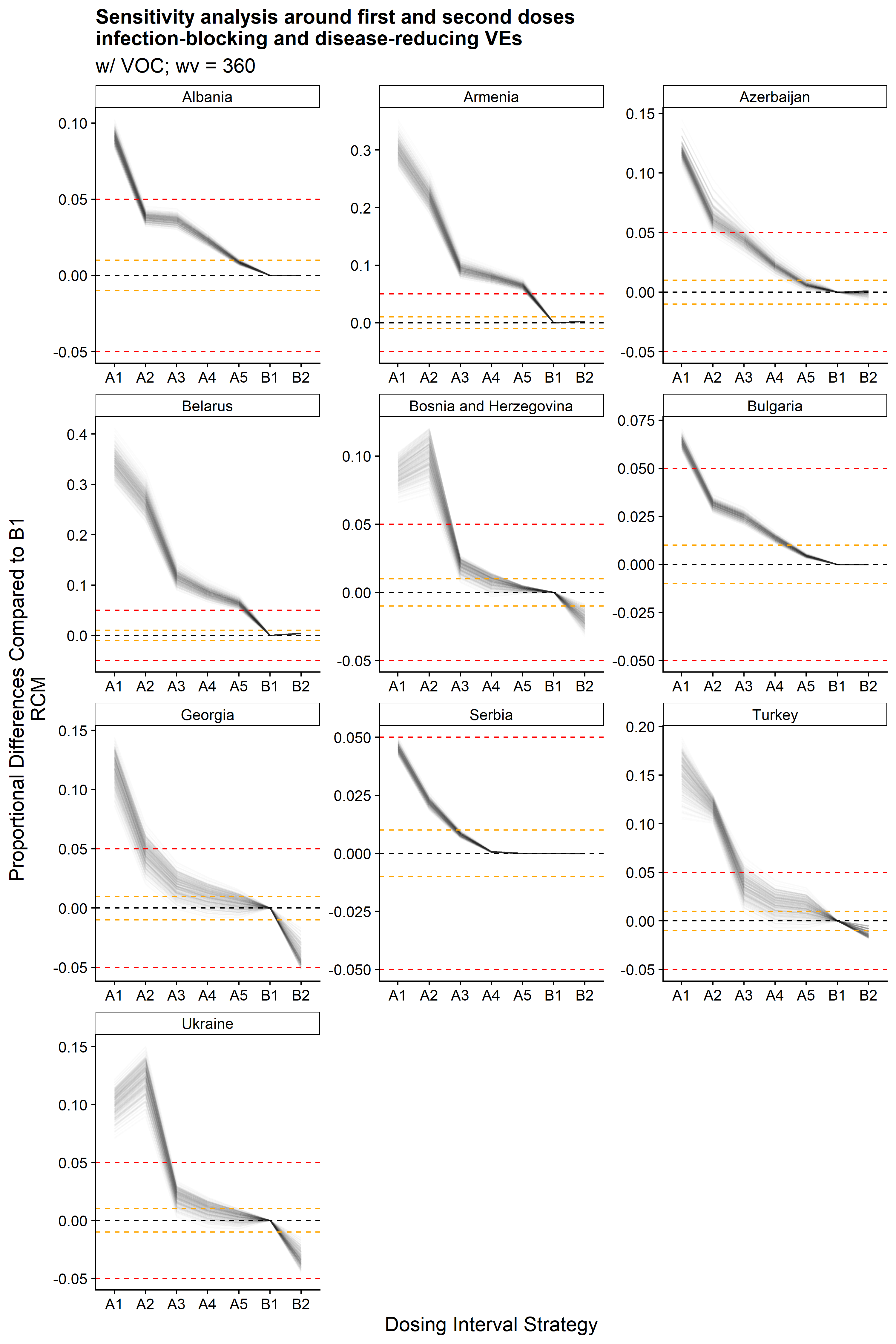
**

### Figure S8. Different outcomes by dosing strategy while accounting for VOC emergence

Strategies A1-A5 and B1-B2 are arranged broadly based on mean effective dosing intervals. Results per strategy for each outcome are scaled to the highest value per country. The number of hospitalisation was used as a proxy for the number of cases with severe disease. The infection hoslitalisation ratio used to calculate this proxy was extract from Salje et al.[(34)](https://sciwheel.com/work/citation?ids=8898230&pre=&suf=&sa=0&dbf=0) Note that these values are relative measures to their respective public health measure – having higher relative measure in deaths than hospitalisations does not mean there are more deaths than hospitalisations, but only that the relatively changes in deaths compared to strategy A1 is greater.


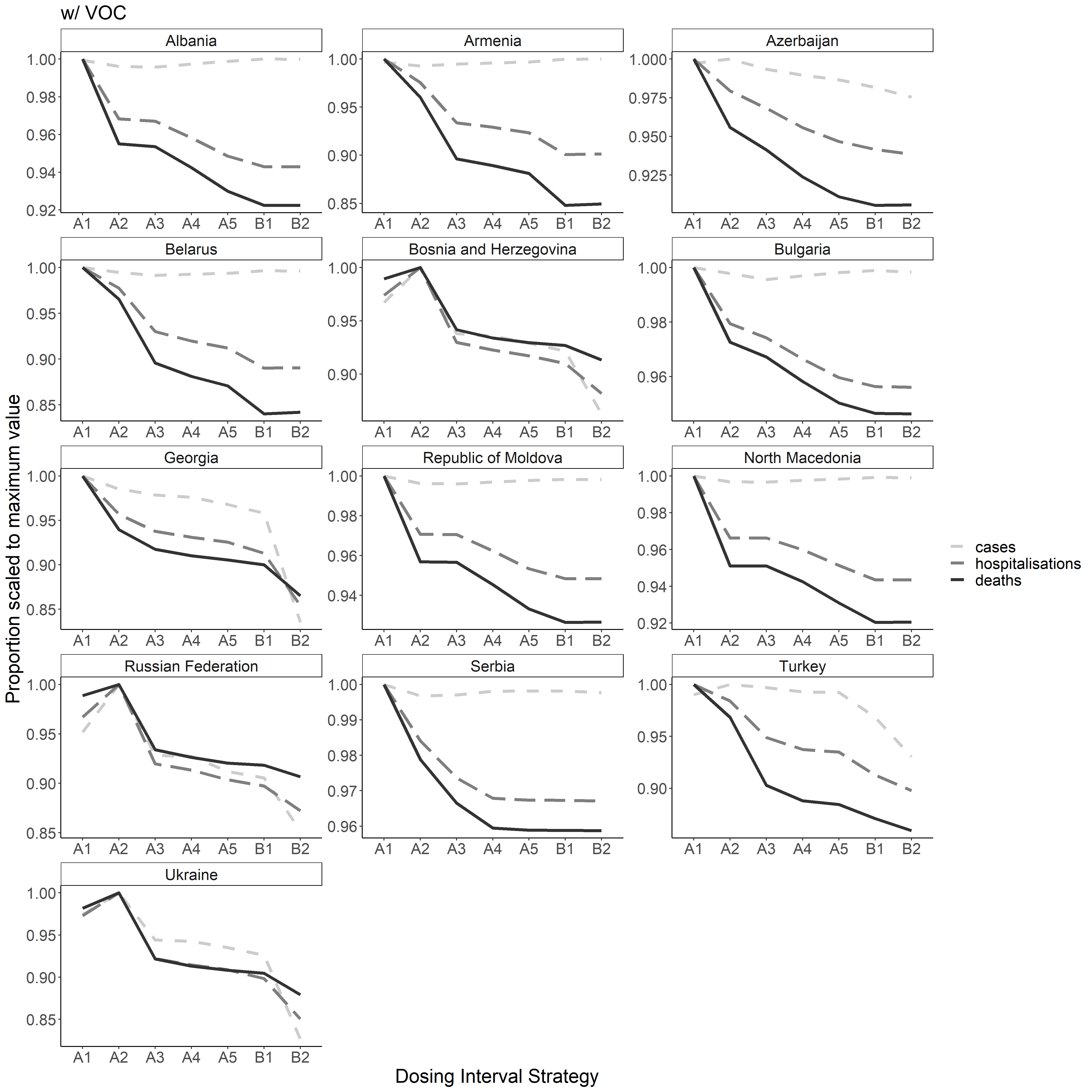


### Figure S9. Different outcomes by dosing strategy without accounting for VOC emergence

Strategies A1-A5 and B1-B2 are arranged broadly based on mean effective dosing intervals. Results per strategy for each outcome are scaled to the highest value per country. The number of hospitalisation was used as a proxy for the number of cases with severe disease. The infection hoslitalisation ratio used to calculate this proxy was extract from Salje et al.[(34)](https://sciwheel.com/work/citation?ids=8898230&pre=&suf=&sa=0&dbf=0) Note that these values are relative measures to their respective public health measure – having higher relative measure in deaths than hospitalisations does not mean there are more deaths than hospitalisations, but only that the relatively changes in deaths compared to strategy A1 is greater.


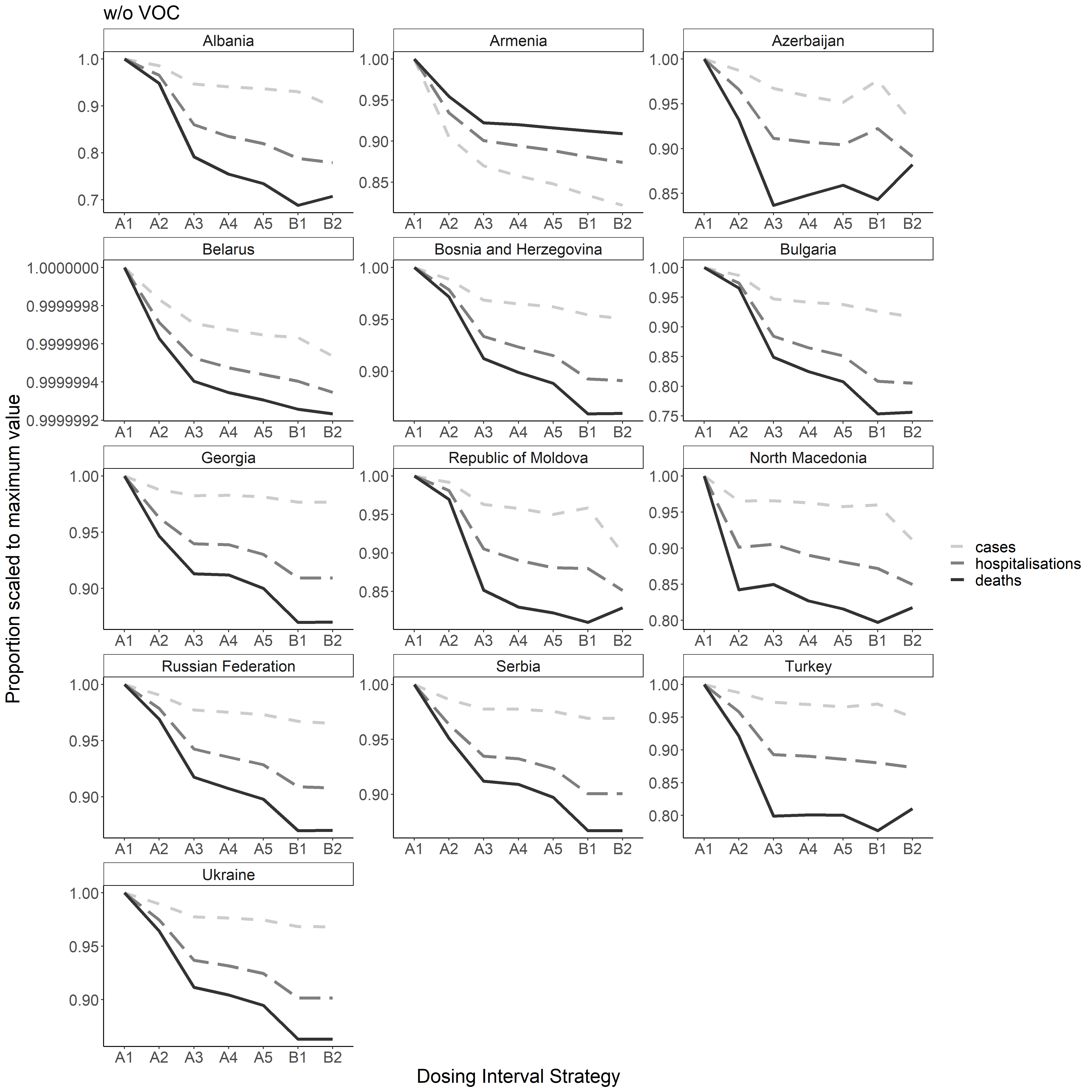


###

# Supplemental Methods

## Population contact patterns

We used observed community mobility to scale population contact patterns (`work` and `other` settings)[(14)](https://sciwheel.com/work/citation?ids=11703648&pre=&suf=&sa=0) using a set of parameters fitted as a part of another study[(5)](https://sciwheel.com/work/citation?ids=10245997&pre=&suf=&sa=0&dbf=0) ‒ significantly reduced community mobility is associated with relative reductions of contacts in the `work` and `other` settings. We used school-related non-pharmaceutical interventions recorded in the OxCGRT (Oxford Covid-19 Government Response Tracker) database to scale the contact patterns in the `school` setting[(35)](https://sciwheel.com/work/citation?ids=11703703&pre=&suf=&sa=0) while keeping `home`-based contacts consistent with the pre-pandemic level.

While projecting into series not included in the fitting process, we assume the COVID-19 stringency index[(35)](https://sciwheel.com/work/citation?ids=11703703&pre=&suf=&sa=0) (strictness of lockdown style policies that primarily restrict people’s behaviour) would gradually return to a near-pre-pandemic level to capture potential long-term behaviour change. The community mobility and population contact patterns would return to near-pre-pandemic levels accordingly, using a Gaussian Generalized Additive Model (GAM) fitted to observations. Contacts in `school` settings follow school terms in the WHO European Region.

More technical details on the assumptions on the population contact patterns are described in Liu et al.[(36)](https://sciwheel.com/work/citation?ids=12467362&pre=&suf=&sa=0&dbf=0)

## Calculating COVID-19 mortality and hospitalisation

This process has been previously described in Liu et al.

1. Infection to mortality delay function is assumed to be a gamma distribution with a mean of 26 (days) and a shape of 5. This probability density function is capped at 60 (days).[(6)](https://sciwheel.com/work/citation?ids=12468223&pre=&suf=&sa=0&dbf=0)

$$P =Gamma(mean = 26; shape = 5)$$

2. The deaths that occurred on day $t$ due to infection on day $t-d$ can be expressed as (where ${IFR}_{j}$ depicts infection fatality ratio among age group $j$).

$${D(t)}_{dj}={E(t-d)}_{j}\cdot P(d)\cdot{IFR}_{j}$$

3. The total deaths occurred on day t can thus be expressed as:

$$D(t) = \sum_{j = 1}^{J} \sum_{d = 1}^{60} {E(t-d)}_{i}\cdot P(d)\cdot{IFR}_{j}$$

## Setting up the sensitivity analyses around the vaccine efficacies

1. We created exhaustively the combinations of infection-reducing VE by first (*ve_i_)* and second doses (*v2e_i_*) and the disease-reducing VE for the first (*ve_d_*) and second doses (*v2e_d_*) by varying them between 0.25 and 0.95, with 0.1 increments; (4096 combinations)
2. We imposed rules that the vaccine efficacies of the first dose do not exceed the second dose (i.e., *ve_i_* ≤ *v2e_i_* and *ve_d_* ≤ *v2e_d_*); (1296 combinations)
3. We imposed rules that the infection-blocking vaccine efficacies do not exceed the disease-reducing vaccine efficacies (*ve_i_* ≤ *ve_d_* and *v2e_i_* ≤ *v2e_d_*); (540 combinations)

## TREND checklist

| **Paper Section/Topic** | **Item No.** | | **Descriptor** | Reported? | | Notes |
| --- | --- | --- | --- | --- | --- | --- |
|  |  |  |  |  | Pg # |  |
| **TITLE and ABSTRACT** | | | |  |  |  |
| Title and Abstract | 1 | | - Information on how units were allocated to interventions |  | Not Applicable | No intervention implemented, mentioned in title it’s a modelling study |
|  |  | | - Structured abstract recommended | ✓ | 2 |  |
|  |  | | - Information on target population or study sample | ✓ | 5-6 |  |
| **INTRODUCTION** | | | |  |  |  |
| Background | 2 | - Scientific background and explanation of rationale | | ✓ | 3 |  |
|  |  | - Theories used in designing behavioral interventions | |  | Not Applicable | No intervention involved |
| **METHODS** | | | |  |  |  |
| Participants | 3 | | - Eligibility criteria for participants, including criteria at different levels in recruitment/sampling plan (e.g., cities, clinics, subjects) | ✓ | 6 | We included criteria we used for including countries in analysis |
|  |  | | - Method of recruitment (e.g., referral, self-selection), including the sampling method if a systematic sampling plan was implemented | ✓ | 5-6, 19 | While we have not done any explicit sampling in this study, we discussed the sampling methods/ biases used by our data sources |
|  |  | | - Recruitment setting |  | Not Applicable | No recruitment involved |
|  |  | | - Settings and locations where the data were collected | ✓ | 5-6, Supplemental Tables S1 and S3 |  |
| Interventions | 4 | | - Details of the interventions intended for each study condition and how and when they were actually administered, specifically including: |  |  |  |
|  |  | | - - Content: what was given? | ✓ | 6-11 | Scenario definition |
|  |  | | - - Delivery method: how was the content given? |  | Not Applicable | No delivery occurred. |
|  |  | | - - Unit of delivery: how were subjects grouped during delivery? | ✓ | 6-11 | We tested a series of dosing interval strategies under different pathogen conditions and epidemic context. Individuals in the simulation model was grouped by age, country, and infection status (i.e. compartments) |
|  |  | | - - Deliverer: who delivered the intervention? |  | Not Applicable | No delivery occurred. |
|  |  | | - - Setting: where was the intervention delivered? |  | Not Applicable | No delivery occurred. |
|  |  | | - - Exposure quantity and duration: how many sessions or episodes or events were intended to be delivered? How long were they intended to last? | ✓ | 6-11 | One or two vaccination events in the simulated population using the hypothetical vaccine (based on AZD 1222). |
|  |  | | - - Time span: how long was it intended to take to deliver the intervention to each unit? | ✓ | 7 | The hypothetical vaccine (based on AZD 1222) would take 14 days to take effect. |
|  |  | | - - Activities to increase compliance or adherence (e.g., incentives) |  | Not Applicable |  |
| Objectives | 5 | | - Specific objectives and hypotheses | ✓ | 3-4 |  |
| Outcomes | 6 | | - Clearly defined primary and secondary outcome measures | ✓ | 3-4 | Cumulative mortality and benefit-risk ratios |
|  |  | | - Methods used to collect data and any methods used to enhance the quality of measurements | ✓ | 4-11, Supplemental Tables S1 and S3 |  |
|  |  | | - Information on validated instruments such as psychometric and biometric properties |  | Not Applicable | No existing instrument used. |
| Sample size | 7 | | - How sample size was determined and, when applicable, explanation of any interim analyses and stopping rules | ✓ | 6 | Sample size not explicitly calculated – it’s where data availability and quality allow for reasonable inference in the geographic region of interests. |
| Assignment method | 8 | | - Unit of assignment (the unit being assigned to study condition, e.g., individual, group, community) | ✓ | 4-5 |  |
|  |  | | - Method used to assign units to study conditions, including details of any restriction (e.g., blocking, stratification, minimization) | ✓ | 5-11 | Dosing interval strategies are used to assign units to vaccination compartments – which is core to the analysis. We have several additional assignment steps in this section. |
|  |  | | - Inclusion of aspects employed to help minimize potential bias induced due to non-randomization (e.g., matching) | ✓ | Supplemental Table S2 | No intervention implemented thus no randomization involved. The vaccination process in the mathematical model relies on expected proportion by compartments as defined in Supplemental Table S2. |
| Blinding (masking) | 9 | | - Whether or not participants, those administering the interventions, and those assessing the outcomes were blinded to study condition assignment; if so, statement regarding how the blinding was accomplished and how it was assessed |  | Not Applicable | There are no participants. |
| Unit of Analysis | 10 | | - Description of the smallest unit that is being analysed to assess intervention effects (e.g., individual, group, or community) | ✓ | 4 | Country-age group. |
|  |  | | - If the unit of analysis differs from the unit of assignment, the analytical method used to account for this (e.g., adjusting the standard error estimates by the design effect or using multilevel analysis) |  | Not Applicable | There is no difference between unit of analysis and unit of assignment – both by country-age group. |
| Statistical methods | 11 | | - Statistical methods used to compare study groups for primary methods outcome(s), including complex methods for correlated data |  | 4-11 | The main analytical method in this study is compartmental transmission model (mathematical model). It doesn’t match the item exactly but we attached the page number. |
|  |  |  | - Statistical methods used for additional analyses, such as subgroup analyses and adjusted analysis |  | Not applicable | We have not used any statistical testing in this study. |
|  |  |  | - Methods for imputing missing data, if used | ✓ | Supplemental methods, p21 | We used linear interpolation for minor missingness in time series data (less than a week consecutively), spatial interpolation and linear regression models for major missingness (borrowing power from neighbours and other WHO/ Europe members) |
|  |  |  | - Statistical software or programs used | ✓ | 11 |  |
| **RESULTS** | | | |  |  |  |
| Participant flow | 12 | | - Flow of participants through each stage of the study: enrollment, assignment, allocation and intervention exposure, follow-up, analysis (a diagram is strongly recommended) |  | Not Applicable | No participants involved. |
|  |  | | - - Enrollment: the numbers of participants screened for eligibility, found to be eligible or not eligible, declined to be enrolled, and enrolled in the study |  | Not Applicable | No participants involved. |
|  |  | | - - Assignment: the numbers of participants assigned to a study condition | ✓ | 4-11 | Simulated populations (by country and age group) were assigned to receive hypothetical vaccine at different timing. |
|  |  | | - - Allocation and intervention exposure: the number of participants assigned to each study condition and the number of participants who received each intervention | ✓ | 9 | The number of vaccines allocated for each country has been defined. |
|  |  | | - - Follow-up: the number of participants who completed the follow-up or did not complete the follow-up (i.e., lost to follow-up), by study condition |  | Not Applicable | No participants involved. |
|  |  | | - - Analysis: the number of participants included in or excluded from the main analysis, by study condition |  | Not Applicable | No participants involved. |
|  |  | | - Description of protocol deviations from study as planned, along with reasons |  | Not Applicable | No intervention implemented. All mathematical simulation. |
| Recruitment | 13 | | - Dates defining the periods of recruitment and follow-up |  | Not Applicable | No participants involved and no intervention implemented. All presented as counterfactual using a mathematical model. |
| Baseline data | 14 | | - Baseline demographic and clinical characteristics of participants in each study condition | ✓ | 5 | Baseline and study population are the same. |
|  |  | | - Baseline characteristics for each study condition relevant to specific disease prevention research | ✓ | 5 | Baseline and study population are the same. |
|  |  | | - Baseline comparisons of those lost to follow-up and those retained, overall and by study condition |  | Not Applicable | No participants involved. |
|  |  | | - Comparison between study population at baseline and target population of interest |  | Not Applicable | Baseline and study population are the same. |
| Baseline equivalence | 15 | | - Data on study group equivalence at baseline and statistical methods used to control for baseline differences |  | Not applicable | Baseline and study population are the same. |
| Numbers analyzed | 16 | | - Number of participants (denominator) included in each analysis for each study condition, particularly when the denominators change for different outcomes; statement of the results in absolute numbers when feasible | ✓ | 5, 7 | 13 countries are included in the full analysis. |
|  |  | | - Indication of whether the analysis strategy was “intention to treat” or, if not, description of how non-compliers were treated in the analyses |  | Not applicable | No intervention involved. |
| Outcomes and estimation | 17 | | - For each primary and secondary outcome, a summary of results for each estimation study condition, and the estimated effect size and a confidence interval to indicate the precision |  | 11-16 | Results have been summarised in the section specified. However, as this is not a statistical study, effect size etc are not relevant. |
|  |  | | - Inclusion of null and negative findings |  | Not Applicable |  |
|  |  | | - Inclusion of results from testing pre-specified causal pathways through which the intervention was intended to operate, if any |  | Not Applicable |  |
| Ancillary analyses | 18 | | - Summary of other analyses performed, including subgroup or restricted analyses, indicating which are pre-specified or exploratory |  | Not Applicable |  |
| Adverse events | 19 | | - Summary of all important adverse events or unintended effects in each study condition (including summary measures, effect size estimates, and confidence intervals) |  | Not applicable | No intervention implemented. |
| **DISCUSSION** | | | |  |  |  |
| Interpretation | 20 | | - Interpretation of the results, taking into account study hypotheses, sources of potential bias, imprecision of measures, multiplicative analyses, and other limitations or weaknesses of the study | ✓ | 17-19 |  |
|  |  | | - Discussion of results taking into account the mechanism by which the intervention was intended to work (causal pathways) or alternative mechanisms or explanations | ✓ | 17-19 | Presented where relevant. |
|  |  | | - Discussion of the success of and barriers to implementing the intervention, fidelity of implementation |  | Not applicable | No intervention implemented. |
|  |  | | - Discussion of research, programmatic, or policy implications | ✓ | 17-19 |  |
| Generalizability | 21 | | - Generalizability (external validity) of the trial findings, taking into account the study population, the characteristics of the intervention, length of follow-up, incentives, compliance rates, specific sites/settings involved in the study, and other contextual issues | ✓ | 17-19 | Presented where relevant, given no intervention implemented. |
| Overall evidence | 22 | | - General interpretation of the results in the context of current evidence and current theory | ✓ | 17-19 |  |

*From:*  Des Jarlais, D. C., Lyles, C., Crepaz, N., & the Trend Group (2004). Improving the reporting quality of nonrandomized evaluations of behavioral and public health interventions: The TREND statement. *American Journal of Public Health*, 94, 361-366. For more information, visit: <http://www.cdc.gov/trendstatement/>

[Bibliography](https://sciwheel.com/work/bibliography?atCursor=false)

[1.    Davies NG, Klepac P, Liu Y, Prem K, Jit M, CMMID COVID-19 working group, et al. Age-dependent effects in the transmission and control of COVID-19 epidemics. Nat Med. 2020 Aug;26(8):1205–11.](https://sciwheel.com/work/bibliography/9102939)

[2.    Levin AT, Hanage WP, Owusu-Boaitey N, Cochran KB, Walsh SP, Meyerowitz-Katz G. Assessing the age specificity of infection fatality rates for COVID-19: systematic review, meta-analysis, and public policy implications. Eur J Epidemiol. 2020 Dec 8;35(12):1123–38.](https://sciwheel.com/work/bibliography/10252041)

[3.    Prem K, Zandvoort K van, Klepac P, Eggo RM, Davies NG, Centre for the Mathematical Modelling of Infectious Diseases COVID-19 Working Group, et al. Projecting contact matrices in 177 geographical regions: An update and comparison with empirical data for the COVID-19 era. PLoS Comput Biol. 2021 Jul 26;17(7):e1009098.](https://sciwheel.com/work/bibliography/11703857)

[4.    United Nations. 2019 World Population Prospects - United Nations Department of Economic and Social Affairs, Population Division [Internet]. 2019 [cited 2020 Mar 17]. Available from: https://population.un.org/wpp](https://sciwheel.com/work/bibliography/8436701)

[5.    Davies NG, Barnard RC, Jarvis CI, Russell TW, Semple MG, Jit M, et al. Association of tiered restrictions and a second lockdown with COVID-19 deaths and hospital admissions in England: a modelling study. Lancet Infect Dis. 2021 Apr;21(4):482–92.](https://sciwheel.com/work/bibliography/10245997)

[6.    Pearson CAB, Bozzani F, Procter SR, Davies NG, Huda M, Jensen HT, et al. COVID-19 vaccination in Sindh Province, Pakistan: A modelling study of health impact and cost-effectiveness. PLoS Med. 2021 Oct 4;18(10):e1003815.](https://sciwheel.com/work/bibliography/12468223)

[7.    Davies NG, Kucharski AJ, Eggo RM, Gimma A, Edmunds WJ, Centre for the Mathematical Modelling of Infectious Diseases COVID-19 working group. Effects of non-pharmaceutical interventions on COVID-19 cases, deaths, and demand for hospital services in the UK: a modelling study. Lancet Public Health. 2020 Jul;5(7):e375–85.](https://sciwheel.com/work/bibliography/9011259)

[8.    Bi Q, Wu Y, Mei S, Ye C, Zou X, Zhang Z, et al. Epidemiology and transmission of COVID-19 in 391 cases and 1286 of their close contacts in Shenzhen, China: a retrospective cohort study. Lancet Infect Dis. 2020 Aug;20(8):911–9.](https://sciwheel.com/work/bibliography/8792615)

[9.    Liu Y, Centre for Mathematical Modelling of Infectious Diseases nCoV Working Group, Funk S, Flasche S. The contribution of pre-symptomatic infection to the transmission dynamics of COVID-2019. [version 1; peer review: 1 approved]. Wellcome Open Res. 2020 Apr 1;5:58.](https://sciwheel.com/work/bibliography/8738168)

[10.   Linton NM, Kobayashi T, Yang Y, Hayashi K, Akhmetzhanov AR, Jung S-M, et al. Incubation Period and Other Epidemiological Characteristics of 2019 Novel Coronavirus Infections with Right Truncation: A Statistical Analysis of Publicly Available Case Data. J Clin Med. 2020 Feb 17;9(2).](https://sciwheel.com/work/bibliography/8415907)

[11.   Nishiura H, Linton NM, Akhmetzhanov AR. Serial interval of novel coronavirus (COVID-19) infections. Int J Infect Dis. 2020 Apr;93:284–6.](https://sciwheel.com/work/bibliography/8415585)

[12.   Hall VJ, Foulkes S, Charlett A, Atti A, Monk EJM, Simmons R, et al. SARS-CoV-2 infection rates of antibody-positive compared with antibody-negative health-care workers in England: a large, multicentre, prospective cohort study (SIREN). Lancet. 2021 Apr 17;397(10283):1459–69.](https://sciwheel.com/work/bibliography/10879406)

[13.   Ritchie H, Mathieu E, Rodés-Guirao L, Appel C, Giattino C, Ortiz-Ospina E, et al. Coronavirus Pandemic (COVID-19) [Internet]. 2020 [cited 2021 Apr 11]. Available from: https://ourworldindata.org/covid-deaths](https://sciwheel.com/work/bibliography/11703906)

[14.   Google. COVID-19 Community Mobility Reports [Internet]. 2021 [cited 2021 May 18]. Available from: https://www.google.com/covid19/mobility/](https://sciwheel.com/work/bibliography/11703648)

[15.   Hale T, Angrist N, Goldszmidt R, Kira B, Petherick A, Phillips T, et al. A global panel database of pandemic policies (Oxford COVID-19 Government Response Tracker). Nat Hum Behav. 2021 Apr;5(4):529–38.](https://sciwheel.com/work/bibliography/10678231)

[16.   Voysey M, Costa Clemens SA, Madhi SA, Weckx LY, Folegatti PM, Aley PK, et al. Single-dose administration and the influence of the timing of the booster dose on immunogenicity and efficacy of ChAdOx1 nCoV-19 (AZD1222) vaccine: a pooled analysis of four randomised trials. Lancet. 2021 Mar 6;397(10277):881–91.](https://sciwheel.com/work/bibliography/10525817)

[17.   Gavi. The COVAX Facility: Interim Distribution Forecast – latest as of 3 February 2021 [Internet]. 2021 [cited 2021 Mar 26]. Available from: https://www.gavi.org/sites/default/files/covid/covax/COVAX-Interim-Distribution-Forecast.pdf](https://sciwheel.com/work/bibliography/11703883)

[18.   WHO. COVAX Announces additional deals to access promising COVID-19 vaccine candidates; plans global rollout starting Q1 2021 [Internet]. 2021 [cited 2021 Apr 11]. Available from: https://www.who.int/news/item/18-12-2020-covax-announces-additional-deals-to-access-promising-covid-19-vaccine-candidates-plans-global-rollout-starting-q1-2021](https://sciwheel.com/work/bibliography/11703893)

[19.   WHO. COVAX reaches over 100 economies, 42 days after first international delivery [Internet]. 2021 [cited 2021 Apr 11]. Available from: https://www.who.int/news/item/08-04-2021-covax-reaches-over-100-economies-42-days-after-first-international-delivery](https://sciwheel.com/work/bibliography/11703889)

[20.   Wouters OJ, Shadlen KC, Salcher-Konrad M, Pollard AJ, Larson HJ, Teerawattananon Y, et al. Challenges in ensuring global access to COVID-19 vaccines: production, affordability, allocation, and deployment. Lancet. 2021 Mar 13;397(10278):1023–34.](https://sciwheel.com/work/bibliography/10480992)

[21.   Robinson E, Jones A, Lesser I, Daly M. International estimates of intended uptake and refusal of COVID-19 vaccines: A rapid systematic review and meta-analysis of large nationally representative samples. Vaccine. 2021 Apr 8;39(15):2024–34.](https://sciwheel.com/work/bibliography/11230457)

[22.   Department of Health and Social Care (UK). UK COVID-19 vaccine uptake plan [Internet]. 2021 [cited 2021 Apr 18]. Available from: https://www.gov.uk/government/publications/covid-19-vaccination-uptake-plan/uk-covid-19-vaccine-uptake-plan](https://sciwheel.com/work/bibliography/11703899)

[23.   Barnard RC, Davies NG, Jit M, John Edmunds W. LSHTM: Updated roadmap assessment – prior to delayed Step 4, 7 July 2021 - GOV.UK [Internet]. 2021 [cited 2021 Sep 16]. Available from: https://www.gov.uk/government/publications/lshtm-updated-roadmap-assessment-prior-to-delayed-step-4-7-july-2021](https://sciwheel.com/work/bibliography/11702792)

[24.   Davies NG, Abbott S, Barnard RC, Jarvis CI, Kucharski AJ, Munday JD, et al. Estimated transmissibility and impact of SARS-CoV-2 lineage B.1.1.7 in England. Science. 2021 Apr 9;372(6538).](https://sciwheel.com/work/bibliography/10593953)

[25.   Mishra S, Mindermann S, Sharma M, Whittaker C, Mellan TA, Wilton T, et al. Changing composition of SARS-CoV-2 lineages and rise of Delta variant in England. EClinicalMedicine. 2021 Sep;39:101064.](https://sciwheel.com/work/bibliography/11695059)

[26.   Shrotri M, Krutikov M, Palmer T, Giddings R, Azmi B, Subbarao S, et al. Vaccine effectiveness of the first dose of ChAdOx1 nCoV-19 and BNT162b2 against SARS-CoV-2 infection in residents of long-term care facilities in England (VIVALDI): a prospective cohort study. Lancet Infect Dis. 2021 Nov;21(11):1529–38.](https://sciwheel.com/work/bibliography/11407622)

[27.   Pritchard E, Matthews PC, Stoesser N, Eyre DW, Gethings O, Vihta K-D, et al. Impact of vaccination on new SARS-CoV-2 infections in the United Kingdom. Nat Med. 2021 Aug;27(8):1370–8.](https://sciwheel.com/work/bibliography/11163054)

[28.   Lopez Bernal J, Andrews N, Gower C, Robertson C, Stowe J, Tessier E, et al. Effectiveness of the Pfizer-BioNTech and Oxford-AstraZeneca vaccines on covid-19 related symptoms, hospital admissions, and mortality in older adults in England: test negative case-control study. BMJ. 2021 May 13;373:n1088.](https://sciwheel.com/work/bibliography/11033114)

[29.   Vasileiou E, Simpson CR, Shi T, Kerr S, Agrawal U, Akbari A, et al. Interim findings from first-dose mass COVID-19 vaccination roll-out and COVID-19 hospital admissions in Scotland: a national prospective cohort study. Lancet. 2021 May 1;397(10285):1646–57.](https://sciwheel.com/work/bibliography/10948526)

[30.   Ismail S, Vilaplana T, Elgohari S, Stowe J, Tessier E, Andrews N, et al. Effectiveness of BNT162b2 mRNA and ChAdOx1 adenovirus vectorCOVID-19 vaccines on risk of hospitalisation among older adults in England: an observational study using surveillance data [Internet]. 2021 [cited 2021 Jul 15]. Available from: https://khub.net/documents/135939561/430986542/Effectiveness+of+BNT162b2+mRNA+and+ChAdOx1+adenovirus+vector+COVID-19+vaccines+on+risk+of+hospitalisation+among+older+adults+in+England.pdf/9e18c525-dde6-5ee4-1537-91427798686b](https://sciwheel.com/work/bibliography/11708384)

[31.   Bernal JL, Andrews N, Gower C, Stowe J, Tessier E, Simmons R, et al. Effectiveness of BNT162b2 mRNA vaccine and ChAdOx1 adenovirus vector vaccine on mortality following COVID-19. medRxiv. 2021 May 18;](https://sciwheel.com/work/bibliography/11120613)

[32.   Harris R, Hall J, Zaidi A, Andrews N, Dunbar K, Dabrera G. Impact of vaccination on household transmission of SARS-COV-2 in England [Internet]. 2021 [cited 2021 Jul 15]. Available from: https://khub.net/documents/135939561/390853656/Impact+of+vaccination+on+household+transmission+of+SARS-COV-2+in+England.pdf/35bf4bb1-6ade-d3eb-a39e-9c9b25a8122a?t=1619601878136](https://sciwheel.com/work/bibliography/11708395)

[33.   Voysey M, Clemens SAC, Madhi SA, Weckx LY, Folegatti PM, Aley PK, et al. Safety and efficacy of the ChAdOx1 nCoV-19 vaccine (AZD1222) against SARS-CoV-2: an interim analysis of four randomised controlled trials in Brazil, South Africa, and the UK. Lancet. 2021 Jan 9;397(10269):99–111.](https://sciwheel.com/work/bibliography/10138638)

[34.   Salje H, Tran Kiem C, Lefrancq N, Courtejoie N, Bosetti P, Paireau J, et al. Estimating the burden of SARS-CoV-2 in France. Science. 2020 Jul 10;369(6500):208–11.](https://sciwheel.com/work/bibliography/8898230)

[35.   Blavatnik School of Government, University of Oxford. Oxford Covid-19 Government Response Tracker (OxCGRT) [Internet]. 2021 [cited 2021 Sep 17]. Available from: https://covidtracker.bsg.ox.ac.uk/](https://sciwheel.com/work/bibliography/11703703)

[36.   Liu Y, Sandmann FG, Barnard RC, Pearson CAB, Pastore R, Pebody R, et al. Optimising health and economic impacts of COVID-19 vaccine prioritisation strategies in the WHO European Region: a mathematical modelling study. Lancet Reg Health Eur. 2022 Jan;12:100267.](https://sciwheel.com/work/bibliography/12467362)
