# Supplementary material for: Dosing interval strategies for two-dose COVID-19 vaccination in 13 middle-income countries of Europe: Health impact modelling and benefit-risk analysis
Source: Lancet Reg Health Eur. 2022 Apr 11;17:100381. doi: 10.1016/j.lanepe.2022.100381 (PMC8996067; doi:10.1016/j.lanepe.2022.100381)
Supplement: Supplementary file 2 [file mmc2.docx]

**Supplemental Tables**

Table S1. Model Parameters

Table S2. Model Description

Table S3. More details on the rationale behind vaccine effectiveness estimates

Table S4. Summary statistics of effective dosing intervals under B1 and B2 given different vaccine supply delays

**Supplemental Figures**

Figure S1. Vaccine roll-out progress among LMICs of the WHO European Regions (n = 20)

Figure S2. Effective dosing interval given 12 weeks of supply delay under strategies B1 and B2

Figure S3. Effective dosing interval given 52 weeks of supply delay under strategies B1 and B2

Figure S4. Relative performance of dosing interval strategies given alternative supply delay levels

Figure S5. Sensitivity analysis using different waning duration (60 and 90 days) by country

Figure S6. Outcomes using a dynamic relationship between dosing interval and the vaccine efficacy achievable after both doses

Figure S7. Sensitivity analyses around four dimensions of vaccine efficacy (infection- and disease-reducing vaccine efficacy after first and second doses)

Figure S8. Different outcomes by dosing strategy while accounting for VOC emergence

Figure S9. Different outcomes by dosing strategy without accounting for VOC emergence

**Supplemental Methods**

Population contact patterns

Calculating COVID-19 mortality and hospitalisation

Setting up the sensitivity analyses around the vaccine efficacies

TREND checklist
